# Insulin-like growth factor binding protein-3 (IGFBP-3): a biomarker of coronary artery disease induced myocardial ischaemia

**DOI:** 10.1093/ehjopen/oeaf028

**Published:** 2025-03-20

**Authors:** Jacqui A Lee, Jessika Wise, Sara D Raudsepp, Louise N Paton, Jan Powell, Kieran Jina, Sally Aldous, Richard W Troughton, Philip D Adamson, A Mark Richards, W Frank Peacock, James L Januzzi, Noah Erceg, Luca Koechlin, Jasper Boeddinghaus, Pedro Lopez-Ayala, Christian Mueller, Martin P Than, John W Pickering, Chris J Pemberton

**Affiliations:** Department of Medicine, Christchurch Heart Institute, University of Otago, Christchurch, Riccarton Avenue, PO Box 4345, Christchurch 8011, New Zealand; Upstream Medical Technologies, 3 Saint Aubyn Street, Devenport, Auckland 0624, New Zealand; Upstream Medical Technologies, 3 Saint Aubyn Street, Devenport, Auckland 0624, New Zealand; Department of Medicine, Christchurch Heart Institute, University of Otago, Christchurch, Riccarton Avenue, PO Box 4345, Christchurch 8011, New Zealand; Department of Medicine, Christchurch Heart Institute, University of Otago, Christchurch, Riccarton Avenue, PO Box 4345, Christchurch 8011, New Zealand; Upstream Medical Technologies, 3 Saint Aubyn Street, Devenport, Auckland 0624, New Zealand; Upstream Medical Technologies, 3 Saint Aubyn Street, Devenport, Auckland 0624, New Zealand; Department of Emergency Medicine, Health NZ-Te Whatu Ora, Christchurch Hospital, 2 Riccarton Avenue, Christchurch 8011, New Zealand; Department of Medicine, Christchurch Heart Institute, University of Otago, Christchurch, Riccarton Avenue, PO Box 4345, Christchurch 8011, New Zealand; Department of Medicine, Christchurch Heart Institute, University of Otago, Christchurch, Riccarton Avenue, PO Box 4345, Christchurch 8011, New Zealand; Department of Medicine, Christchurch Heart Institute, University of Otago, Christchurch, Riccarton Avenue, PO Box 4345, Christchurch 8011, New Zealand; Henry JN Taub Department of Emergency Medicine, Baylor College of Medicine, 15 Taub Loop, Houston, TX 77030, USA; Harvard Medical School & Division of Cardiology, Massachusetts General Hospital, Baim Institute for Clinical Research, 930 Commonwealth Avenue #3, Boston, MA 02215, USA; Cardiovascular Research Institute Basel (CRIB) and Department of Cardiology, University Hospital Basel, Spitalstrasse 2, 4056 Basel, Switzerland; Cardiovascular Research Institute Basel (CRIB) and Department of Cardiology, University Hospital Basel, Spitalstrasse 2, 4056 Basel, Switzerland; Cardiovascular Research Institute Basel (CRIB) and Department of Cardiology, University Hospital Basel, Spitalstrasse 2, 4056 Basel, Switzerland; Cardiovascular Research Institute Basel (CRIB) and Department of Cardiology, University Hospital Basel, Spitalstrasse 2, 4056 Basel, Switzerland; Cardiovascular Research Institute Basel (CRIB) and Department of Cardiology, University Hospital Basel, Spitalstrasse 2, 4056 Basel, Switzerland; Department of Emergency Medicine, Health NZ-Te Whatu Ora, Christchurch Hospital, 2 Riccarton Avenue, Christchurch 8011, New Zealand; Department of Emergency Medicine, University of Kansas Medical Center, 3901 Rainbow Boulevard, Kansas City, KS 66160, USA; Department of Medicine, University of Otago, Christchurch, 2 Riccarton Avenue, Christchurch 8011, New Zealand; Department of Medicine, Christchurch Heart Institute, University of Otago, Christchurch, Riccarton Avenue, PO Box 4345, Christchurch 8011, New Zealand; Department of Emergency Medicine, Health NZ-Te Whatu Ora, Christchurch Hospital, 2 Riccarton Avenue, Christchurch 8011, New Zealand; Department of Medicine, Christchurch Heart Institute, University of Otago, Christchurch, Riccarton Avenue, PO Box 4345, Christchurch 8011, New Zealand; Upstream Medical Technologies, 3 Saint Aubyn Street, Devenport, Auckland 0624, New Zealand

**Keywords:** Biomarker, IGFBP-3, Troponin, Imaging, Acute coronary syndromes, CAD

## Abstract

**Aims:**

Among individuals presenting to the emergency department (ED) with chest pain, clinical uncertainty surrounds the appropriate identification of non-myocardial infarction (MI) individuals who would most benefit from objective functional/anatomical testing (e.g. imaging). We applied a proteomic biomarker discovery approach to identify novel candidates reflecting coronary artery disease (CAD) induced ischaemia that could translate to measurement in clinical samples.

**Methods and results:**

Mass spectroscopy (MS) of perfusate from an isolated rat heart model of cardiac ischaemia identified >100 novel protein biomarkers. A prominent candidate, insulin-like growth factor binding protein (IGFBP-3), was then interrogated for its ability to identify CAD-related ischaemia (e.g. positive cardiac stress test; unstable angina pectoris, UAP; arterial stenosis >70% on angiography) in multiple patient sample sets [cardiac stress testing, *n* = 12; septal alcohol ablation (SAA), *n* = 12; ED chest pain, *n* = 2977]. In cardiac stress testing, a significant delta IGFBP-3 (ΔIGFBP-3) between 0 and 150 min was seen in positive, but not negative, tests (*P* = 0.03). In SAA, peripheral IGFBP-3 levels did not change over 24 h (*P* = 0.57). In ED patients, ΔIGFBP-3 between 0 and 2 h (i) identified more 365-day low-risk major adverse cardiac event cases (27–30%), (ii) provided 7% improvement in positive predictive value over a clinical model for the identification of unstable angina (*P* = 0.01), and (iii) was a significant, independent predictor of >70% stenosis on angiography, improving indeterminate risk prediction by 9% (95% CI 3–15%).

**Conclusion:**

Our discovery approach has translated IGFBP-3 as a potential biomarker to identify significant CAD/ischaemia in patients who do not meet diagnostic thresholds for MI.

## Graphical Abstract

**Graphical Abstract:**
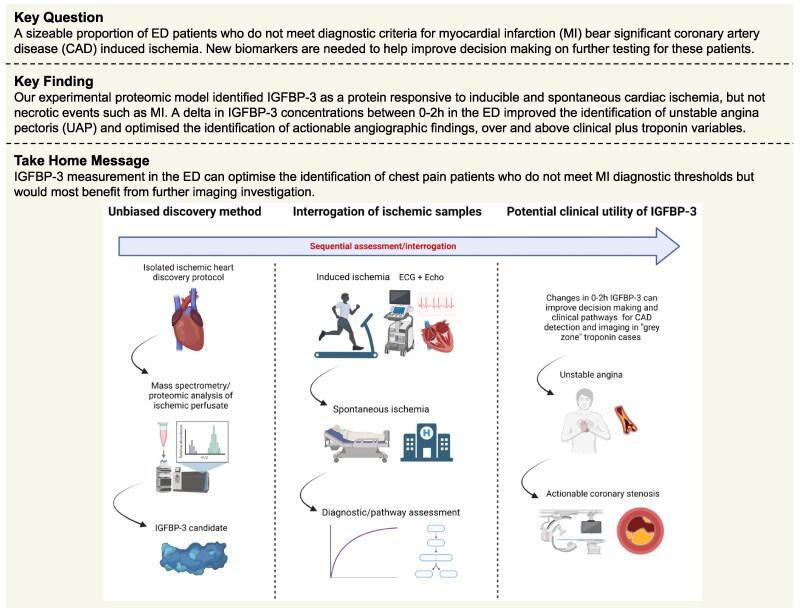

## Introduction

Diagnostic protocols that use high-sensitivity cardiac troponin (hs-cTn) testing are the standard of care for the Emergency Department (ED) assessment of patients presenting with suspected acute coronary syndrome (ACS).^1–4^ A significant proportion of these patients in whom myocardial infarction (MI) is excluded (or ‘ruled out’) have underlying coronary artery disease (CAD) that may lead to adverse cardiac events soon after evaluation. Current guidelines recommend objective functional (stress) or anatomical (imaging) testing for individuals in this group to determine individual patient risk; however, uncertainty remains as to deciding which patients truly benefit from such objective testing. Score-based triaging, e.g. the No Objective Testing (NOT) rule,^5–7^ and consideration of incremental gradients in sub-threshold hs-cTn levels,^8,9^ have been suggested to improve the identification of low-risk individuals. These approaches still leave a sizeable proportion of ED patients (up to one-third) who are not ‘low risk’, have potentially significant CAD, and may still experience post-discharge unstable angina pectoris (UAP).^10,11^ This group of patients represents a major unmet need for biomarker support to aid decision-making beyond the current use of clinical scoring and troponin.

In an effort to identify candidate biomarkers indicative of myocardial ischaemia, we utilized a discovery platform comprising ischaemic isolated heart perfusate, coupled with mass spectrometry (MS) proteomic analyses. In doing so, we identified insulin-like growth factor binding protein-3 (IGFBP-3) as a novel candidate marker of myocardial ischaemia. We then sought to determine the potential of IGFBP-3 to identify myocardial ischaemia by the interrogation of circulating levels in (i) stress testing induced ischaemia, (ii) septal ablation procedures (alcohol-induced myocardial necrosis), (iii) trans-organ gradients obtained during clinical catheterization, and (iv) patients presenting to a hospital ED with suspicion of ACS.

## Methods

### Isolated heart procedure

**Figure 1:**
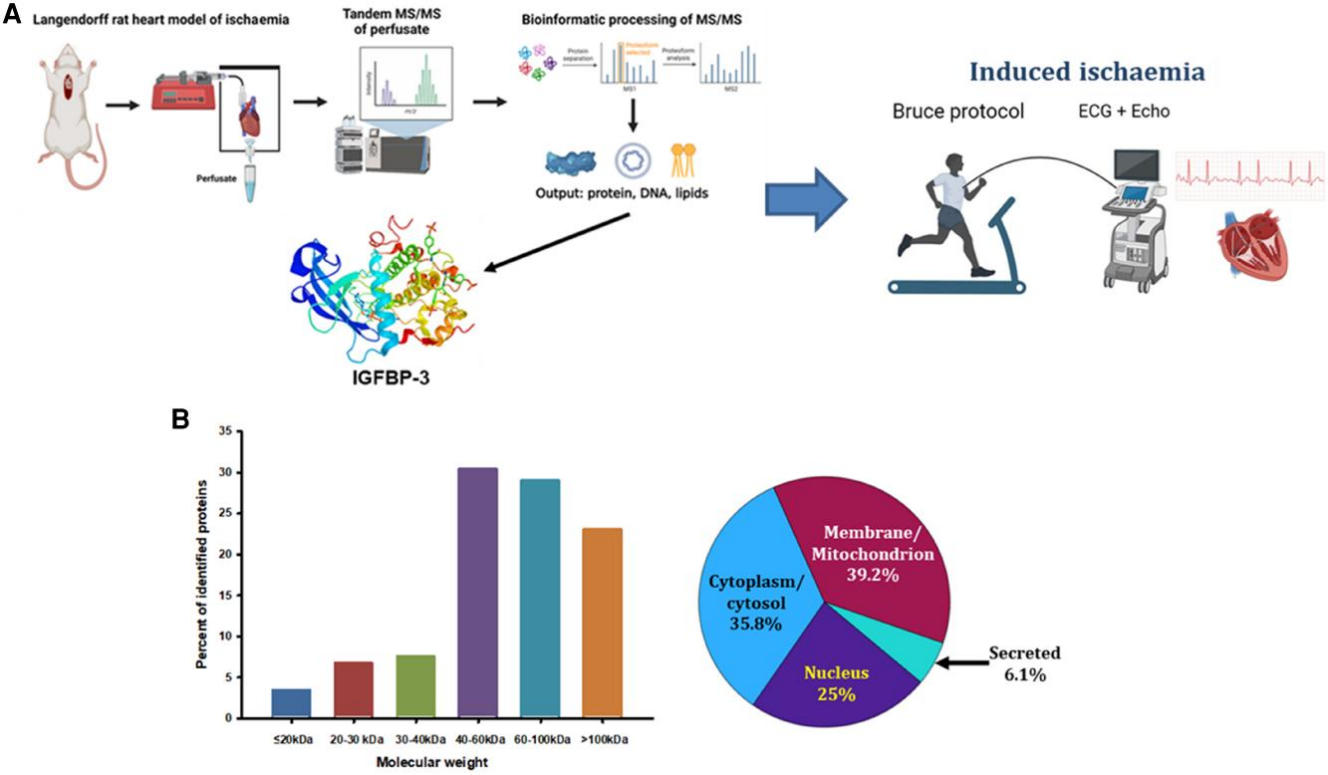
(*A*) Schematic of the experimental ischaemia marker discovery/test process used herein. Perfusate obtained from ischaemic and control isolated rat hearts was subjected to unbiased tandem MS/MS proteomic interrogation. This process identified 148 protein factors as a novel (i.e. presence in ischaemic hearts but not controls) of which insulin-like growth factor binding protein-3 emerged as a readily testable candidate which was then investigated in human experimental induced ischaemia (cardiac exercise stress testing). (*B*) Molecular weight ranges and cellular distributions of the 148 proteins identified in trypsin-digested isolated perfused rat heart perfusates at *t* = 10 min. Only 6% of all identified proteins were predicted to be secreted products, with over half of the proteins between 40 and 100 kDa in size.

Isolated heart experiments were carried out in conformity with the ARRIVE guidelines, after animal ethics committee approval (AUP 21-178). Isolated perfused rat heart (IPRH) preparations were carried out as previously described^12,13^ (see Supplementary Methods). Briefly, ischaemia was induced by reversible and sham ligation of the left main coronary artery in isolated hearts. Perfusate was collected at −10 min prior to, 0, +10, +20, and +30 min after ligation. Reversible and sham perfusates were subjected to unbiased HPLC/MS to detect differences in protein expression between the two sample types (*Figure 1A* and Supplementary Methods).

### Insulin-like growth factor binding protein-3 and high-sensitivity cardiac troponin assays

Full IGFBP-3 assay details and its analytical validation are given in the Supplementary Methods. Briefly, IGFBP-3 levels were determined using a two-antibody sandwich ELISA on plasma samples diluted 1:100 with phosphate-buffered saline (PBS)/albumin assay buffer (pH 7.2), with samples corrected for dilution in all calculations (i.e. plate values were multiplied by 100×). The IGFBP-3 assay has a limit of detection (LoD) of 0.37 ng/mL, a 10% coefficient of variation (CV), a limit of quantification (LoQ) of 1.56 ng/mL, and an inter-assay CV of 8.9 and 7.9% at 6.5 and 23.9 ng/mL, respectively, over 38 consecutive assays. Incurred sample reanalyses (ISRs) revealed 94.5% of tested samples to be within the required limit of 30% of the mean.^14^

Cardiac troponin T (cTn) was measured in all studies using the Roche Cobas e411 assay (hs-cTnT, Roche, Rokruetz, Switzerland) with a limit of blank (LoB) = 3 ng/L, LoQ = 5 ng/L, and a single upper European reference limit (URL) = 14 ng/L. cTnI was measured in APACE and SPACE by the Abbott ARCHITECT (Abbott Park, IL) with a LoB = 0.7 ng/L, LoQ = 3 ng/L, and an overall URL of 26 ng/L. cTnI was measured in the FAST-TRAC study using the Beckman Coulter (Beckman Coulter, Inc, Chaska, MN, USA) assay with a LoB = 0.33 ng/L, LoQ = 3 ng/L, and an overall URL of 18.2 ng/L.

### Clinical studies/plasma sample collection

All human studies were conducted in conformity with the Declaration of Helsinki and all participants gave written informed consent. All data were analysed in conformity with STARD guidelines. Human EDTA plasma samples were obtained from multiple studies as follows: individuals undergoing cardiac exercise stress testing [Signal Peptides in Induced Cardiac Ischaemia (SPICI) study, Christchurch, NZ, *n* = 12], patients undergoing septal ablation treatment^15,16^ [SEARCH study, Christchurch, NZ, *n* = 12], and regional plasma samples from patients undergoing clinically indicated cardiac catheterization from whom matched arterio-venous samples were drawn to obtain trans-organ (venous minus arterial levels of markers) gradients^17,18^ [GRADIENT study, Christchurch, NZ, *n* = 14], and three separate prospective, observational hospital ED studies of patients presenting with chest pain suspicious of ACS [APACE study, international (*n* = 1056),^11,19^ FAST-TRAC, USA (*n* = 1230),^20^ and SPACE, Christchurch, New Zealand (*n* = 766)].^21,22^

### SPICI study

This study consisted of healthy control individuals (*n* = 6), and participants with known CAD (*n* = 6), each undergoing treadmill exercise stress testing as previously described for assessment of possible inducible cardiac ischaemia.^23^ The study was approved by the Health and Disability Ethics Committee (HDEC) of New Zealand (URB/10/03/010). Full details of the protocol are given in the Supplementary Methods.

### SEARCH study

Twelve patients with hypertrophic cardiomyopathy (HOCM),^24^ and scheduled for septal alcohol ablation (SAA) from January 2021 to April 2023, were consented and enrolled in this study, approved by the HDEC of New Zealand (14STH73). Details of the protocol are given in the Supplementary Methods. We collected venous blood samples for determination of plasma biomarkers in EDTA-filled tubes 10 min before the procedure and at 15, 30, 45, 60, 120, 240, and 1440 min (24 h) after induction of myocardial necrosis.

### GRADIENT study

Patients (*n* = 14) undergoing cardiac catheterisation for diagnostic coronary angiography or percutaneous intervention were enrolled and gave written consent for additional blood sampling as previously described.^25,26^ The study was approved by the HDEC of New Zealand (URB/06/02/010). The right femoral artery and vein were cannulated with 6 Fr sheaths under local anaesthesia. A multipurpose, diagnostic catheter (6 Fr), was advanced via the femoral vein to draw sequential blood samples from femoral, renal, hepatic and jugular veins, and the pulmonary artery. An Amplatz left-1 (6 Fr) catheter was employed to sample from the cardiac coronary sinus. Blood was drawn from the femoral artery before and after the completion of venous sampling to assess the stability of peptide levels during the time required for sampling. Intravenous saline up to 500 mL was administered through the procedure. Blood samples were collected in EDTA and centrifuged within 20 min at 3000 *g* and 4°C. Plasma was stored at −80°C.

### ED studies

To assess the potential diagnostic/prognostic biomarker ability of IGFBP-3 in patients with possible myocardial ischaemia, we interrogated plasma samples obtained from ED patients presenting with chest pain suspicious of ACS from three separate studies (APACE, FAST-TRAC, and SPACE), all with similar recruitment criteria. Patient demographics and heterogeneity are given in *Table 1*, and details of recruitment criteria are in the Supplementary Methods. In all three ED studies, blood samples were drawn at presentation +1 and +2 h.

**Table 1:** Patient demographics of emergency department studies

Characteristics	All patients	SPACE	FAST-TRAC	APACE
Patients, *n*	3052	766	1230	1056
Age (years)	59 (50–71)	62 (54–73)	56 (48–67)	61 (49–73)
Sex, *n*				
* *Male	1947 (64)	500 (65)	732 (60)	672 (64)
* *Female	1105 (36)	266 (35)	498 (40)	384 (36)
Adjudicated diagnoses				
MI	420 (14)	148 (19)	80 (7)	192 (18)
* *Type 1	355 (12)	139 (18)	68 (6)	148 (14)
* *Type 2	65 (2)	9 (1)	12 (1)	44 (4)
UA	251 (8)	59 (8)	100 (8)	92 (9)
Non-ACS	2381 (78)	569 (73)	1050 (85)	772 (73)
Previous medical history				
* *MI	748 (25)	230 (30)	268 (22)	250 (24)
* *CAD	1057 (35)	301 (39)	423 (34)	333 (32)
* *Diabetes	567 (19)	114 (15)	261 (21)	192 (18)
* *Hypertension	1841 (60)	452 (59)	775 (63)	614 (58)
* *Cholesterol	1625 (53)	497 (65)	632 (51)	496 (47)
* *PCI	772 (25)	226 (30)	281 (23)	265 (25)
* *CABG	220 (7)	57 (7)	87 (7)	76 (7)
Presentation medicines				
* *Aspirin	1466 (48)	519 (68)	545 (44)	402 (38)
* *Dual anti-platelet	520 (17)	218 (28)	169 (14)	133 (13)
* *ACEi/ARB	1489 (49)	293 (38)	741 (60)	455 (43)
* *Beta-blocker	1506 (49)	333 (43)	794 (65)	379 (36)
ECG results				
Abnormal	826 (27)	200 (26)	330 (27)	296 (28)
* *Ischaemia	517 (17)	164 (21)	148 (12)	205 (19)
* *ST-segment elevation	95 (3)	16 (2)	47 (4)	32 (3)
Physiology				
* *Heart rate (bpm)	75 (65–87)	70 (61–81)	78 (68–90)	76 (66–88)
* *SBP (mmHg)	140 (125–157)	142 (127–162)	140 (125–157)	139 (124–154)
* *DBP (mmHg)	80 (70–89)	80 (70–90)	80 (71–90)	79 (70–89)
Respiration (min^−1^)	17 (16–20)	16 (15–18)	18 (16–20)	16 (14–19)
* *BMI	27.8 (24.5–31.6)	28.3 (25.1–31.7)	28.5 (24.8–32.9)	26.8 (23.7–30.0)
* *O_2_ sat (%)	98 (96–99)	97 (96–98)	98 (97–99)	98 (96–99)
Clin. chemistry, markers				
* *Hb (g/L)	140 (129–150)	142 (131–152)	137 (125–148)	142 (131–151)
* *Platelets (×10^9^/L)	232 (193–275)	231 (196–273)	239 (198–287)	224 (188–266)
* *eGFR-EPi (mL/min 1.73 m^2^)	72 (57–90)	61 (52–70)	87 (87)	86 (70–100)
* *Na^+^ (mmol/L)	139 (138–141)	139 (138–141)	139 (137–141)	140 (138–141)
* *K^+^ (mmol/L)	4.0 (3.8–4.3)	4.0 (3.8–4.3)	4.0 (3.7–4.3)	4.0 (3.8–4.3)
Glucose	6.0 (5.3–7.2)	6.1 (5.4–7.2)	5.8 (5.3–7.1)	6.1 (5.4–7.4)
* *hs-TnT, 0 h (ng/L)	8.9 (5–19.2)	8.1 (4.2–18.5)	9.6 (6.0–18.9)	8.0 (4.0–21.0)
* *hs-TnT, 2 h (ng/L)	8.4 (4.7–20.3)	8.2 (4.2–19.9)	8.4 (5.1–18.8)	9.0 (4.0–22.0)
* *hs-TnI, 0 h (ng/L)	4.0 (2.3–13.6)	4.5 (2.0–14.5)	3.7 (2.3–11.2)	4.0 (2.0–15.2)
* *hs-TnI, 2 h (ng/L)	4.3 (2.3–16.0)	5.3 (2.7–18.3)	3.6 (2.3–13.0)	4.3 (2.0–19.0)
* *IGFBP-3, 0 h (ng/mL)	1587 (1226–1976)	1316 (1007–1620)	1482 (1157–1816)	1858 (1465–2271)
* *IGFBP-3, 2 h (ng/mL)	1480 (1138–1880)	1211 (943–1480)	1445 (1134–1753)	1854 (1431–2241)

### Primary endpoints

The primary endpoints for analyses among patients presenting with possible ACS were the diagnosis of UAP, the angiographic finding of obstructive CAD ≥70% in at least one coronary artery, qualification for NOT rule inclusion, and major adverse cardiac events (MACEs; defined as new ACS or CV death) within 365 days of presentation (including any index event).

### Adjudication of final diagnoses

Adjudication of the final diagnosis was performed centrally for each study individually by two independent cardiologists, with a third serving as a tiebreaker in the case of diagnostic disagreement. The gold standard diagnosis was determined by a review of all available medical records through locally applied follow-up.

STEMI, NSTEMI, and UAP were defined per the fourth Universal Definition of MI.^27^ NSTEMI was diagnosed when there was evidence of acute myocardial necrosis (i.e. a rise and/or fall in cTn, with at least one measurement >99th percentile) in a clinical presentation consistent with ACS but without ST-segment elevations on ECG. In cases that included significant new and persistent ST-segment elevations on ECG together with myocardial necrosis, STEMI was diagnosed. The following criteria were used to indicate an increased likelihood of a diagnosis of UA: typical angina pectoris at rest; worsening/deterioration of previously stable angina; cardiac stress test showing myocardial ischaemia; coronary angiography revealing a diameter stenosis of at least 70%; fractional flow reserve documenting the functional significance of a coronary lesion; and sudden cardiac death or MI occurred during follow-up.^17,28^

### NOT rule calculation and 365-day MACE assessment

Allocation of patients into NOT rule categories was carried out in accordance with previous reports.^5–7^ Briefly, patients were considered eligible for NOT rule inclusion if MI was ruled out according to current protocols, defined as hs-cTn levels at 0–2 h below thresholds (<14 ng/L Roche hs-TnT) with no ischaemic changes on the ECG. The NOT rule was only calculated on hs-TnT as that is the only consistent hs-cTn assay across all three studies. Eligible NOT rule patients were then classified into ‘low risk’ (<3 risk factors, age <50 years) and ‘not low risk’ (history of CAD or MI) as previously descibed.^28^ MACE was defined as ACS (MI or UAP) or cardiovascular death within 365 days of presentation, including index events.

### Statistics

Experimental IPRH hemodynamic data are presented as mean ± standard error of the mean. Results are presented as median with interquartile range (IQR). All statistical analysis was carried out using SPSS (IBM/SPSS, v29). Categorical variables were described as percentages. Relational analysis of plasma protein/peptide concentrations was done using Spearman rank order correlation testing and linear regression analysis. The *t*-test, Mann–Whitney, and χ^2^ tests were used as appropriate to compare patient characteristics. In ED patient studies, binary logistic regression was used to build multivariable clinical and biomarker models that could be incorporated into receiver operating characteristic (ROC) curves to investigate the usefulness of each variable and their combinations for the diagnosis of UAP or the presence of 70% coronary stenosis. Base models were created using clinical characteristics and hs-TnT. IGFBP-3 and ΔIGFBP-3 were then added to the model to evaluate their ability to improve the ROC characteristics [AUC, specificity, sensitivity, positive predictive value (PPV), and NPV]. Pathways and decision curve analyses were used to determine patient movement across risk profiles that were attributable to IGFBP-3.

## Results

### Identification of IGFBP-3 in ischaemic isolated rat heart perfusate

Coronary ligation resulted in frank changes in hemodynamics in the ischaemic isolated rat heart (IIRH) model, not seen in sham/control hearts (see Supplementary material online, *Figure S1A*). MS analysis of trypsin-digested isolated heart perfusates identified 148 unique proteins including IGFBP-3 (*Figure 1A*) as present in samples taken at 10 min post-ligation, but not in sham/control samples. The distribution of these proteins revealed that 35.8% had a cytoplasmic/cytosolic location, 25% were nuclear, 39.2% membranous/mitochondrial, and 6.1% secretory (*Figure 1B*). Of the secreted proteins, IGFBP-3 was identified via two high-quality, deconvoluted peptide sequences (see Supplementary material online, *Figure S1B*).

### Response of circulating IGFBP-3 to induced cardiac ischaemia in humans

**Figure 2:**
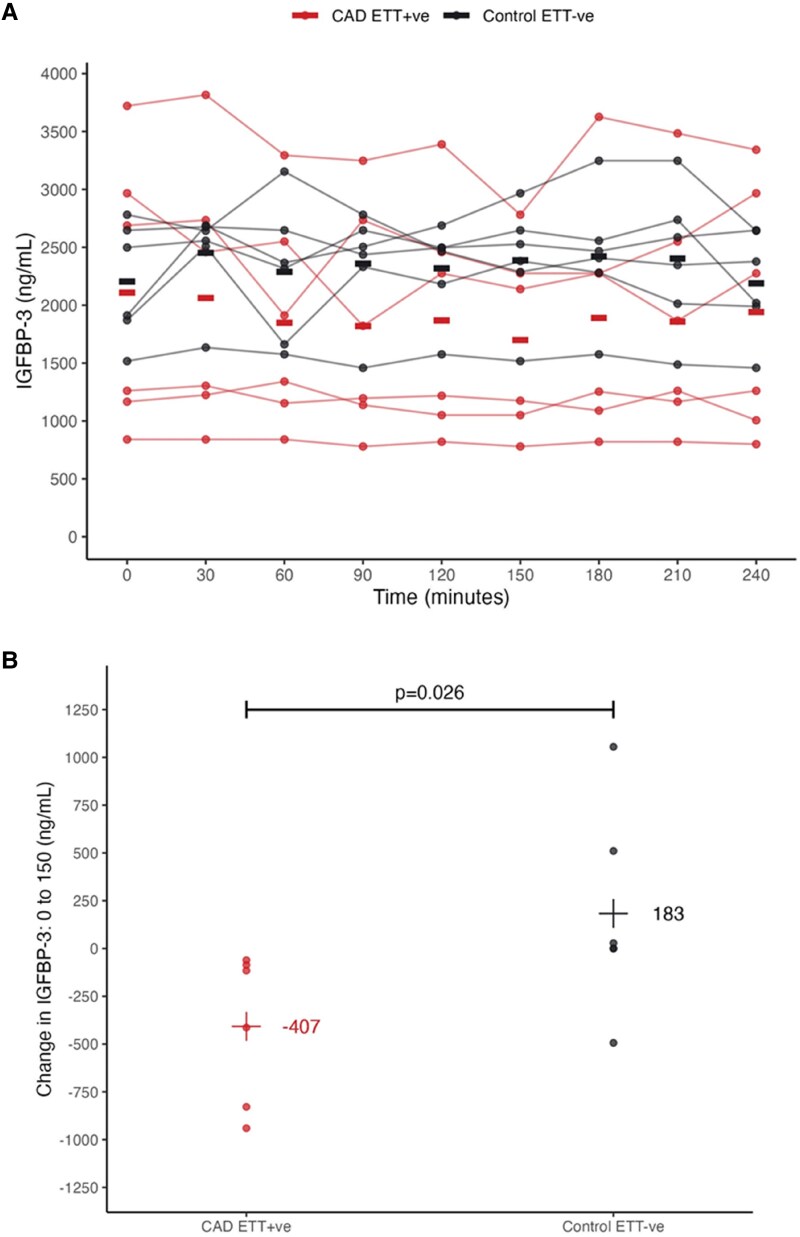
(*A*) Mean (horizontal bars) and individual circulating levels of insulin-like growth factor binding protein-3 in control stress test negative (*n* = 6, black) and CAD stress test positive (*n* = 6, red) cases across 240 min. Stress testing was initiated at *t* = 0 using a standard Bruce protocol with echo imaging to confirm ischaemia. (*B*) Mean 0–150 min (delta) insulin-like growth factor binding protein-3 (ΔIGFBP-3) changes in CAD stress test positive cases (CAD positive EST) were negative, compared with positive in control negative EST cases at 150 min (*P* = 0.026).

IGFBP-3, as measured by our assay, was confirmed by Western Blot as consistent with the 44–46 kDa molecular weight doublet forms that are well described (see Supplementary material online, *Figure S2*).^29,30^ With the identification of IGFBP-3 as a responsive factor in an experimental model of cardiac ischaemia, we undertook an assessment of the response of circulating IGFBP-3 in human inducible cardiac ischaemia (patient demographics, Supplementary material online, *Table S1*). In healthy volunteers, with no evidence of CAD and negative stress tests, mean plasma IGFBP-3 levels across the measured time period tended to rise compared with *t* = 0 values (black triangles, *n* = 6, *Figure 2A*). In contrast, in patients with known CAD and positive stress tests, mean plasma IGFBP-3 levels decreased to nadirs at 90 and 150 min post-stress (red squares, *n* = 6, *Figure 2A*). Of note, the mean 0–150 min shift in IGFBP-3 (ΔIGFBP-3, defined as the 150 min value minus the 0 min value) in positive stress test cases was −407 ± 392 ng/mL vs. +183 ± 532 ng/mL in negative stress test cases (*Figure 2B*, Mann–Whitney U test *P* = 0.03). Pre-test mean hs-cTnT concentrations in positive stress test cases (22.6 ± 39 ng/L) tended to be higher than control (3.7 ± 1.8 ng/L, *P* = 0.27). The mean 0–150 min delta of hs-TnT (3.8 ± 3.1 ng/L) in positive EST cases was not significant compared with negatives (*P* = 0.87, data not shown).

### Regional trans-organ levels of IGFBP-3 and response to a septal alcohol ablation model of human cardiac necrosis

**Figure 3:**
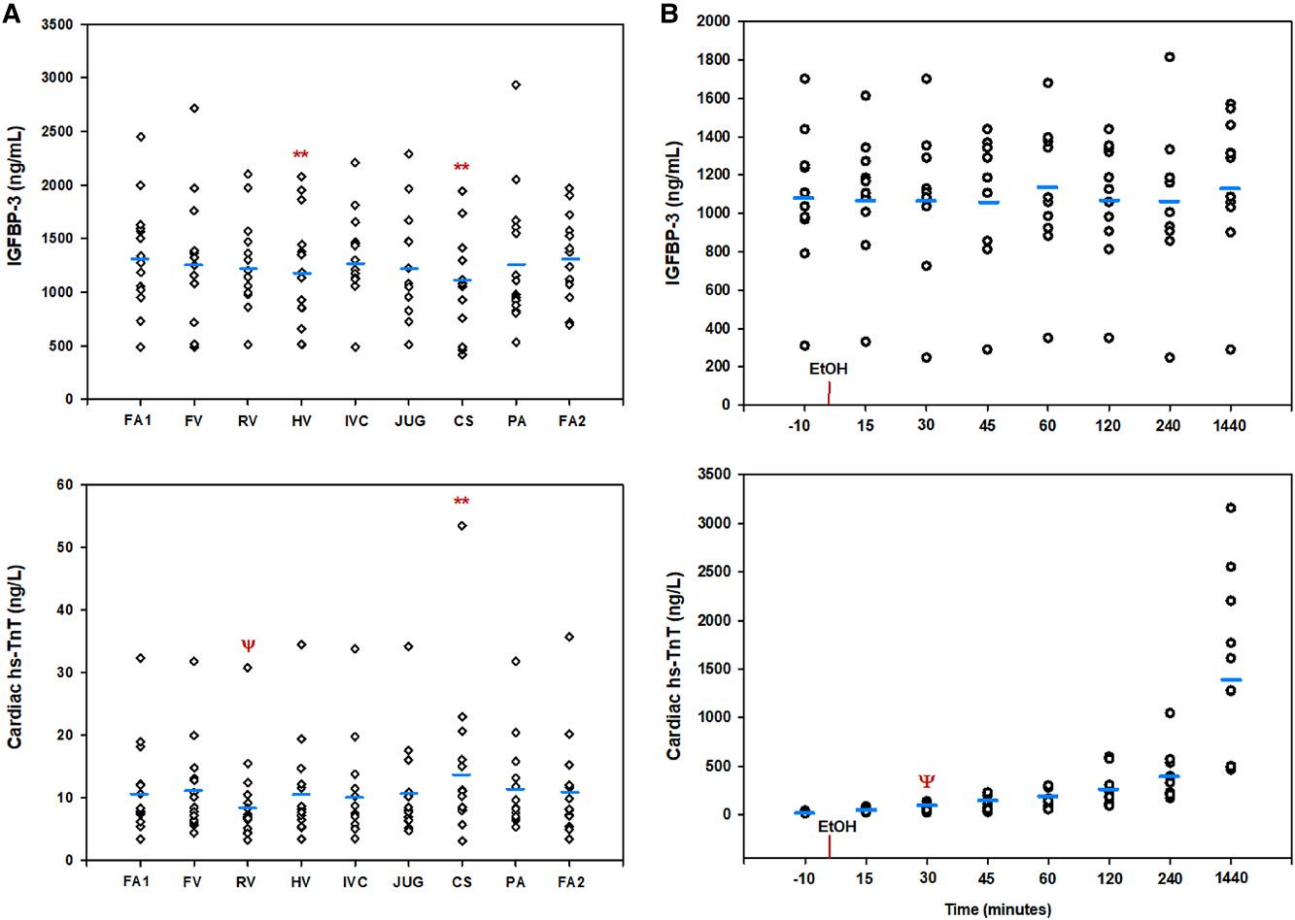
(*A*) Mean (blue line) and individual insulin-like growth factor binding protein-3 (upper panel) and hs-TnT (lower panel) concentrations across multiple arterio-venous organ beds (*n* = 14). FA1, entry arterial; FA2, exit arterial; FV, femoral vein; RV, renal vein; HV, hepatic vein; IVC, inferior vena cava; JUG, jugular; CS, cardiac coronary sinus; PA, pulmonary artery. Due to the time taken for sampling and to account for cannulation effects, arterio-venous comparisons were made with time-comparable sites, i.e. CS vs. FA2 and RV/HV vs. FA1. Extraction was noted across both CS and HV sites for insulin-like growth factor binding protein-3, whereas for hs-TnT, there was production across the heart and extraction across the kidney. Red ** *P* < 0.05, Ψ = *P* < 0.01. (*B*) Mean insulin-like growth factor binding protein-3 (upper panel) and hs-TnT (lower panel) levels in response to septal alcohol ablation (*n* = 12). Whereas IGFBP-3 levels showed no real change, hs-TnT levels rose significantly from 30 min post-ablation (*P* = 0.01, red Ψ) and continued to rise out to 1440 min (24 h).

In order to better define circulating changes in IGFBP-3, its interaction with the heart (and other organs), and its response to more severe cardiac ischaemia, we undertook trans-organ arterio-venous profiling of IGFBP-3. We documented IGFBP-3 temporal response across human localized alcohol-induced myocardial necrosis (demographics of both groups are in Supplementary material online, *Table S2*). In 14 patients undergoing cardiac catheterization with regional blood sampling, mean coronary sinus (CS) levels of IGFBP-3 were lower than paired arterial levels (1067 ± 449 ng/mL vs. 1314 ± 400 ng/mL, *P* = 0.01), indicating net cardiac extraction/degradation of IGFBP-3 (*Figure 3A*). A similar negative gradient was observed across the hepatic circulation (mean hepatic IGFBP-3 = 1194 ± 515 ng/mL vs. mean arterial = 1343 ± 508 ng/mL, *P* = 0.03). There was no evidence of other trans-organ gradients for IGFBP-3. Mean hs-cTnT concentrations displayed net cardiac release (13.1 ± 12.3 ng/L vs. arterial 10.1 ± 8.2 ng/L, *P* = 0.02) and renal extraction/degradation (mean renal = 8.6 ± 6.9 ng/L vs. arterial 10.1 ±7.6 ng/L, *P* = 0.002, *Figure 3A*).

In 12 patients undergoing SAA (a clinical ‘model’ of MI), IGFBP-3 levels were not significantly altered by the intervention, with no ΔIGFBP-3 observed from 0 to 15 min, nor out to 1440 min (24 h), after injection of ethanol into the selected septal artery (*Figure 3B*). In contrast, hs-cTnT levels rose markedly from *t* = 15 min post-ethanol (*P* = 0.009, *t* = 15 min vs. 10 min) and continued to rise out to 24 h post-procedure (*Figure 3B*). We do not have data on the effect of time between balloon stenosis and the introduction of ethanol.

### Diagnostic performance of IGFBP-3 in acute chest pain emergency department patients with MI ruled out

**Figure 4:**
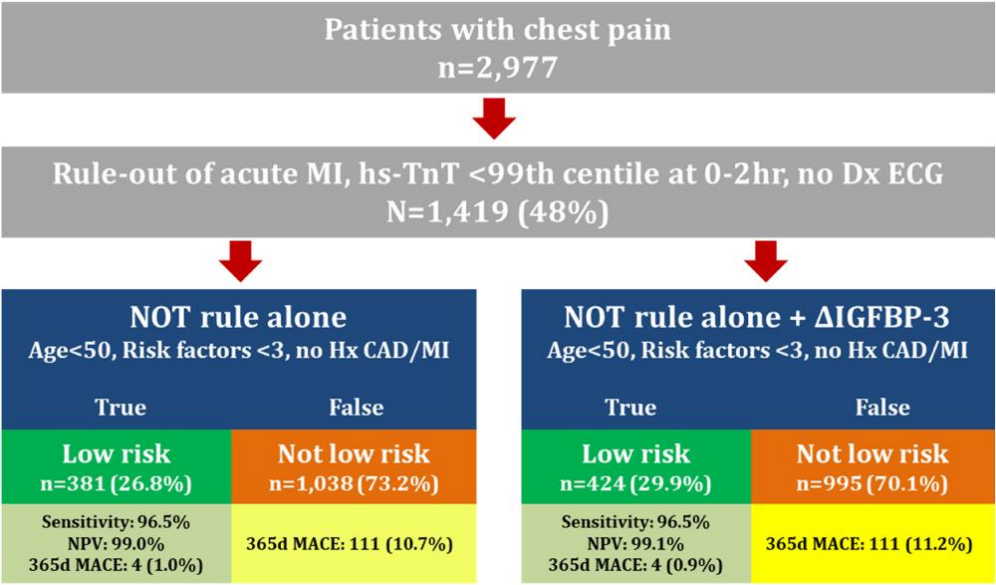
Assessment of the impact of ΔIGFBP-3 in the emergency department upon the No Objective Testing rule calculations in all emergency department studies combined. A total of 1419 patients were eligible for the No Objective Testing rule inclusion, of which the No Objective Testing rule calculations determined *n* = 381 (26.8%) were low risk with a 365-day major adverse cardiac event rate of 1.0%. The addition of a positive 0–2 h ΔIGFBP-3 increased the absolute number (+43) and proportion of true low-risk cases to *n* = 424 (29.9%), decreasing the 365-day major adverse cardiac event rate to 0.9%.

Based on the results from experimental and human ischaemia studies, we investigated the potential of plasma *t* = 0 IGFBP-3 + ΔIGFBP-3 (defined as *t* = 2 h minus the *t* = 0 h value) measurement to impact clinical diagnoses/prediction of outcomes in ED patients presenting with chest pain, or equivalent symptoms, triggering assessment for possible ACS, with a focus on those in whom MI had been ruled out. Demographic/heterogeneity details of all patients are contained in *Table 1*. Neither presenting IGFBP-3 level nor ΔIGFBP-3 predicted the diagnosis of acute MI in the total dataset, including Type 1 or Type 2 MI (AUC for all MI = 0.53, *n* = 408 cases out of 2901). However, out of 1419 data matched NOT rule eligible cases, the addition of ΔIGFBP-3 identified more low-risk cases, increasing from *n* = 381 (26.8%) to *n* = 424 (29.9%) (proportional difference 3.1%; 95% CI −0.4 to 6.4%, *Figure 4*).

**Figure 5:**
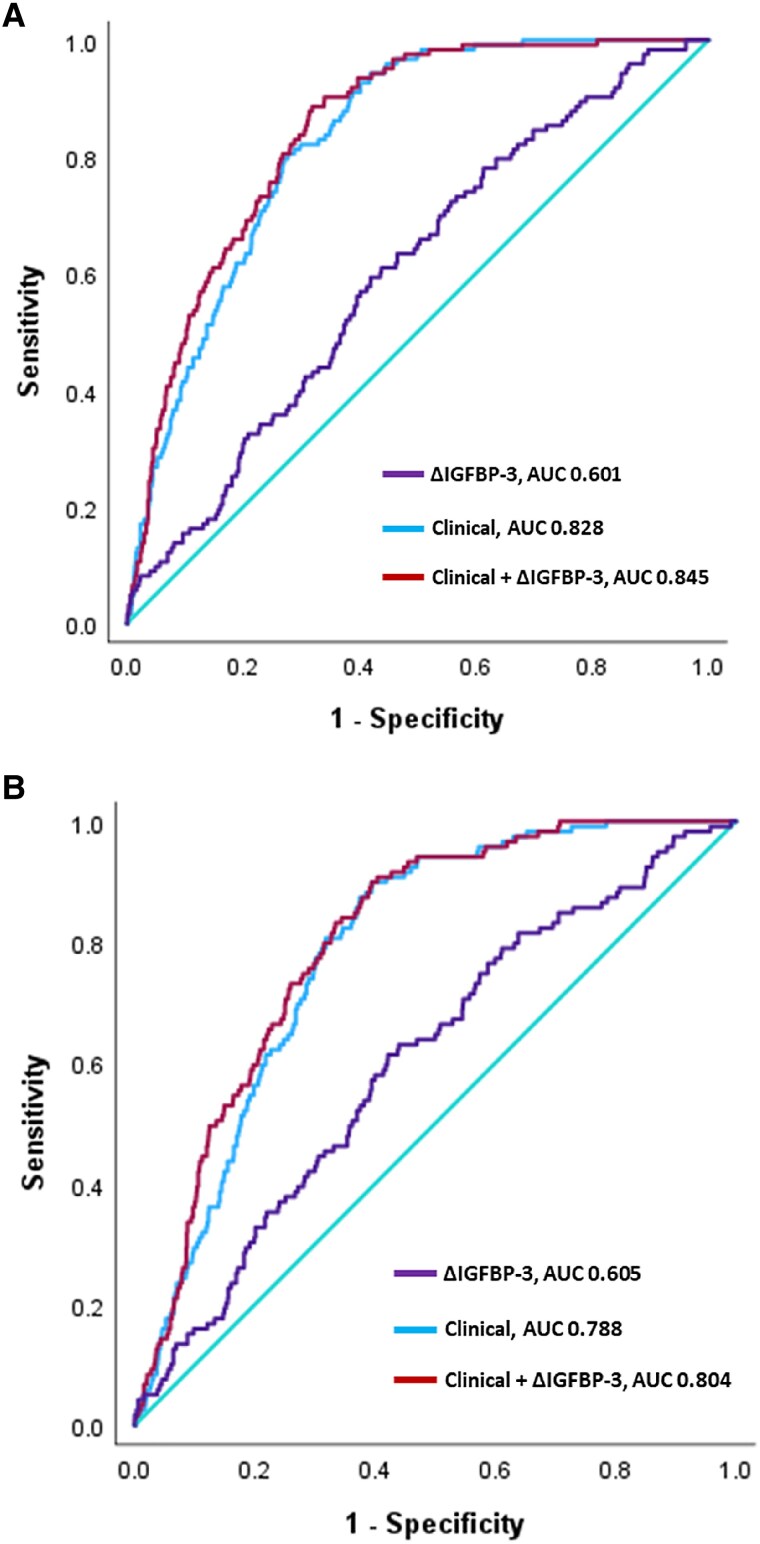
Receiver operating characteristic curves generated by logistic regression models for the diagnosis of unstable angina pectoris in (*A*) all No Objective Testing rule patients (*n* = 1695 cases, 123 unstable angina pectoris) and (*B*) where the 0–2 h hs-TnT maximum was between 5 and 14 ng/L (*n* = 1180 cases, 119 unstable angina pectoris). In both scenarios, a positive ΔIGFBP-3 provided a significant addition to receiver operating characteristic curves (0.012 and *P* = 0.05, respectively) containing a clinical model comprising age, sex, Hx of Cholesterol, Hx CAD/MI, ECG, and Log10 hs-TnT maximum. ΔIGFBP-3 had the most impact at high specificity through improved sensitivity.

Initial assessment in the NOT rule patients from the SPACE study alone revealed median 0–2 h ΔIGFBP-3 values to be elevated in those presenting with adjudicated UAP [*n* = 35, 327 (117–719) ng/mL] vs. all other diagnoses [*n* = 415, −112 (−380–190) ng/mL, *P* < 0.001]. Further, positive 0–2 h ΔIGFBP-3 values were an independent predictor of a UAP diagnosis in a multivariable regression model that included the history of CAD/MI, cholesterol, sex, and age (OR = 1.6; 1.2–2.3, *P* = 0.003). Expanding this analysis to NOT rule eligible patients from all three ED studies (*n* = 1695 total, *n* = 123 UAP), logistic regression modelling revealed ΔIGFBP-3 to improve the AUC of the base clinical model (age, sex, cholesterol, ECG, history of CAD/MI, and the maximum troponin T concentration) from 0.83 to 0.85 (ΔAUC 0.01, 95% CI 0.002–0.02) (*Table 2*). At 90% specificity, ΔIGFBP-3 improved the sensitivity of the ROC from 41.5 to 56.1%, PPV 24.6 to 30.6%, and the NPV from 95.1 to 96.3% (*Figure 5A*, *Table 2*). Similar results were obtained using the same models when the only criterion was that the maximum 0–2 h hs-cTnT concentration was between 5 and 14 ng/L (PPV at 90% specificity increased from 24.8 to 31.7%, *Figure 5B*, *Table 2*, *P* = 0.05). Overall, the addition of ΔIGFBP-3 to these analyses improved the PPV for UAP at 90% specificity across both assessments by 7%. The logistic regression models for both scenarios are given in Supplementary material online, *Table S3* and decision curve analyses in Supplementary material online, *Figure S4*.

**Table 2:** Receiver operating characteristic AUCs, sensitivities (sens), specificities (spec), NPV, and PPV performance data of an emergency department clinical model (age + sex + HxCAD/MI + HxDyslipid + ECG + hsTnTMAX) for the outcomes of diagnosis of unstable angina pectoris and predicting 70% stenosis on angiography in subgroups shown

						Clinical vs. Clinical + IGFBP-3 + ΔIGFBP-3				
Outcome	Subgroup	*n*	*n* with outcome	Model	AUC (95% CI)	ΔAUC	Sensitivity	Specificity	NPV	PPV
All myocardial infarction	All	2901	408 (14.1%)	IGFBP-3 + ΔIGFBP-3	0.53 (0.51–0.57)		—	—	—	—
Unstable angina pectoris	NOT rule eligible	1695	123 (7.3%)	IGFBP-3 + ΔIGFBP-3	0.61 (0.56–0.66)		15.4	90.0	93.1	10.8
				Clinical	0.83 (0.81–0.86)		41.5	90.0	95.1	24.6
				Clinical + IGFBP-3 + ΔIGFBP-3	0.85 (0.82–0.87)	0.01 (0.002 to 0.02)	56.1	90.0	96.3	30.6
Unstable angina pectoris	hsTnTMAX <14	1838	154 (8.4%)	IGFBP-3 + ΔIGFBP-3	0.59 (0.55–0.64)		90.0	15.8	94.5	8.9
				Clinical	0.82 (0.79–0.84)		90.0	60.7	98.5	17.3
				Clinical + IGFBP-3 + ΔIGFBP-3	0.82 (0.80–0.85)	0.007 (−0.001 to 0.02)	90.0	65.9	98.6	19.4
Unstable angina pectoris	hsTnTMAX <14, no HxCAD/MI	1339	50^a^ (3.7%)	IGFBP-3 + ΔIGFBP-3	0.59 (0.45–0.67)		90.0	15.5	97.6	4.0
				Clinical	0.83 (0.77–0.88)		90.0	60.0	99.4	8.0
				Clinical + IGFBP-3 + ΔIGFBP-3	0.83 (0.78–0.88)	0.005 (−0.01 to 0.02)	90.0	59.0	99.3	7.8
Unstable angina pectoris	hsTnTMAX >5 and <14	1180	119 (10.1%)	IGFBP-3 + ΔIGFBP-3	0.61 (0.55–0.66)		16.0	90.0	90.5	15.2
				Clinical	0.79 (0.76–0.82)		29.4	90.0	91.9	24.8
				Clinical + IGFBP-3 + ΔIGFBP-3	0.81 (0.77–0.83)	0.02 (0.001–0.031)	39.5	90.0	93.2	31.7
70% stenosis	NOT rule eligible	193	102 (52.8%)	IGFBP-3 + ΔIGFBP-3	0.65 (0.57–0.71)		90.0	22.2	66.5	56.4
				Clinical	0.72 (0.64–0.79)		90.0	39.6	78.0	62.5
				Clinical + IGFBP-3 + ΔIGFBP-3	0.74 (0.67–0.82)	0.03 (−0.01 to 0.06)	90.0	52.7	82.5	68.0
70% stenosis	hsTnTMAX <14	219	111 (50.7%)	IGFBP-3 + ΔIGFBP-3	0.65 (0.58–0.72)		90.0	22.2	68.2	54.3
				Clinical	0.75 (0.68–0.81)		90.0	38.1	78.7	59.9
				Clinical + IGFBP-3 + ΔIGFBP-3	0.77 (0.71–0.83)	0.03 (−0.007 to 0.06)	90.0	54.6	84.2	67.1
70% stenosis	hsTnTMAX <14, no HxCAD/MI	110	42^a^ (38.2%)	IGFBP-3 + ΔIGFBP-3	0.64 (0.49–0.75)		11.9	90.0	62.3	42.4
				Clinical	0.76 (0.66–0.88)		35.7	90.0	69.4	68.8
				Clinical + IGFBP-3 + ΔIGFBP-3	0.80 (0.70–0.88)	0.04 (−0.002 to 0.10)	57.1	90.0	77.2	77.9
70% stenosis	hsTnTMAX >5 and <14	154	84 (54.5%)	IGFBP-3 + ΔIGFBP-3	0.65 (0.55–0.73)		90.0	20.0	62.5	57.4
				Clinical	0.76 (0.66–0.84)		90.0	28.6	70.5	60.2
				Clinical + IGFBP-3 + ΔIGFBP-3	0.80 (0.72–0.87)	0.04 (0.002 to 0.10)	90.0	48.6	80.3	67.7

The impact of adding ΔIGFBP-3 is given as an increase in AUC (ΔAUC) and *P*-value. Impact analyses were undertaken at 90% sensitivity or 90% specificity. Decision curves relevant to this data are shown in Supplementary material online, *Figure S4*.

^a^Small number of events means the results should be treated with caution because of the possibility of over-fitting.

In NOT rule patients who subsequently underwent cardiac stress testing (EST, *n* = 679), ΔIGFBP-3 values were higher in positive [*n* = 66, 13.1 (−98.9–112.9) ng/mL] vs. negative [*n* = 613, −52.5 (−212.5–108.2) ng/mL] tests (*P* = 0.04). Logistic regression revealed ΔIGFBP-3 to be an independent predictor of a subsequent positive EST [OR = 1.33 (1.02–1.72), *P* = 0.04], adding to the combined predictive abilities of the maximum hs-TnT value [OR = 3.43 (1.11–10.6), *P* = 0.03], and history of CAD/MI [OR = 1.86 (1.09–3.21), *P* = 0.03]. This predictive effect of ΔIGFBP-3 was strongest in those who had no reported history of CAD/MI [*n* = 41, OR = 1.40 (1.02–1.92), *P* = 0.04]. In EST patients whose maximum hs-cTnT value was >5 and <14 (*n* = 470), the predictive ability of ΔIGFBP-3 to detect positive tests [*n* = 54, OR = 1.37 (0.98–1.92), *P* = 0.06] was less strong than hs-TnT [OR = 14.4 (1.12–186), *P* = 0.04] and history of CAD/MI [OR = 1.78 (0.98–3.24), *P* = 0.06]. In those with no history of CAD/MI in this subgroup (*n* = 31), neither the maximum hs-cTnT (*P* = 0.10) or ΔIGFBP-3 (*P* = 0.17) were predictive of positive tests.

**Figure 6:**
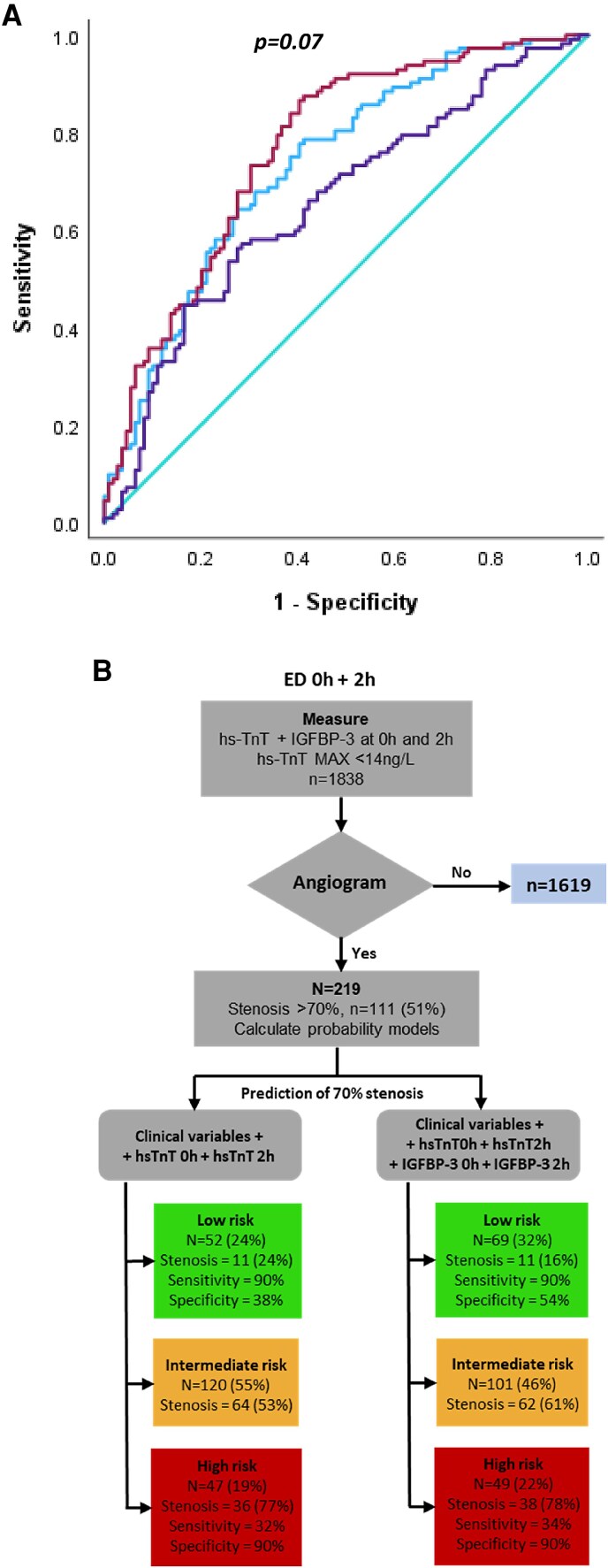
(*A*) Receiver operating characteristic curves and derived data for the findings of 70% stenosis (*n* = 112) on coronary angiography in all cases where 0–2 h hs-TnT < 14 ng/L (*n* = 221). The addition of ΔIGFBP-3 improved the AUC of the clinical + hs-TnT curve by 0.033 points (*P* = 0.07). (*B*) In pathways, analyses of cases in which hs-TnT at *t* = 0 and *t* = 2 h were both <14 ng/L, inclusion of ΔIGFBP-3 for the detection of 70% coronary stenosis (*n* = 219 total) improved the clinical + hs-TnT based identification of low-risk cases by an absolute 8% (*n* = 52, 24% to *n* = 69, 32%), all of which had no 70% stenosis. This was due to improved specificity of 38–54% at 90% sensitivity.

In NOT rule eligible patients and in those with a maximum hs-TnT >5 and <14 (*n* = 154), IGFBP-3 with ΔIGFBP-3 improved the prediction of 70% coronary artery stenosis over clinical variables (ΔAUC 0.03, 95% CI −0.01 to 0.06, and 0.04, 95% CI 0.002–0.10) (*Table 2*). Furthermore, in CA patients with a maximum 0–2 h hs-TnT <14 (*n* = 219), addition of IGFBP-3 improved the ROC AUC to predict 70% stenosis from 0.75 to 0.77 (ΔAUC 0.03, 95% CI −0.007 to 0.06, *Figure 6A*). In pathway analysis of these same CA patients with 0–2 h hs-TnT <14 ng/L, ΔIGFBP-3 allowed the shifting of 19 intermediate risk cases, primarily to low risk (*n* = 17 low risk, *n* = 2 high risk, *Figure 6B*). Thus, an absolute 9% decrease in intermediate risk cases was achieved, all correctly reallocated to low or high risk.

Finally, in matched ED cases with a maximum hs-TnT < 14 and a finding of 70% stenosis on CA (*n* = 150), decreased presentation values of IGFBP-3 alone were a predictor of new ACS events within 365 days (*n* = 16, OR = 0.45; 0.24–0.85), *P* = 0.01), whereas the maximum value of hs-cTnT was not (OR = 0.63; 0.05–8.3, *P* = 0.73).

## Discussion

The main findings of this work are: (i) IGFBP-3 is a protein that responds to the stimulus of coronary ischaemia without necrosis in an isolated cardiac setting, (ii) circulating IGFBP-3 exhibits dynamic shifts during provocative human cardiac EST such that a ΔIGFBP-3 occurs between 0 and 120/150 min in positive EST cases, (iii) levels of IGFBP-3 do not appear to be responsive to a clinical, acute toxic source of cardiac necrosis (alcohol ablation) nor to naturally occurring MI, (iv) IGFBP-3 undergoes net extraction/degradation by heart and liver, (v) a 0–2 h ΔIGFBP-3 in ED chest pain patients has potential to detect cardiac ischaemia in clinical scored/hs-TnT grey zone (5–14 ng/L) cases experiencing UAP at hospital ED presentation, (vi) ΔIGFBP-3 can improve the identification of patients with sub-threshold troponin values who would most benefit from objective imaging, and (vii) in the ED, ΔIGFBP-3 appears to add to current clinical variables, including hs-cTnT measurement.

The isolated perfused heart has previously been used for the experimental proteomic identification of markers of cardiac ischaemia and infarction.^31,32^ However, the vast majority of reports have not taken the further step of attempting to translate findings into human clinical studies. In performing this step, the sheer number of proteins identified herein (*n* = 148), prompted us to carefully consider which to pursue as potential useful markers. Three factors prompted us to investigate IGFBP-3. First, the early (∼10 min) appearance of IGFBP-3 in cardiac perfusates after coronary ligation suggested it may react to ischaemia rather than MI. Second, Koomen *et al*.^31^ also identified IGFBP-3 in rat heart perfusates, validating our discovery, and third, IGFBP-7 has found promise as a useful prognostic marker in heart failure, thus linking the IGFBP family to CVD.^25,33^ The cardiac stress testing and SAA groups were chosen as trial testing groups to determine temporal and biochemical responses of IGFBP-3 to (i) ischaemia without infarction and (ii) a clinical model of infarction where the onset is precisely known. This is a logical step to take after unbiased proteomic analyses of isolated heart perfusate revealed IGFBP-3 as a potential ischaemia marker. Both cardiac stress testing and SAA groups are not intended to be large robust datasets—they are integrated into our overall discovery process to rapidly identify markers that have the potential to be properly investigated (*Figure 1*) in the more robust ED datasets which provide the more invaluable information.

Measurement of circulating IGFBP-3 is clinically routine on multiple automated analysers and is an accepted surrogate marker of growth and GH production, due to it being responsible for the carriage of up to 90% of circulating IGF-I/II.^34^ However, not all immunoassays are equivalent as IGFBP-3 is subject to cleavage by multiple proteases and binds multiple proteins, all of which can affect antibody–antigen binding.^35^ In our IGFBP-3 assay, we instituted a binding enhancement step to reduce protease and binding protein interference to attempt to minimize these effects. Importantly, although there are well-validated normal range values for IGFBP-3 use in assessing disorders of growth,^34,35^ that is not the consideration here; instead, a directional change of IGFBP-3 between 0 and 2 h (i.e. ΔIGFBP-3), regardless of starting value, is what provided potentially useful information.

Preliminary assessment of IGFBP-3 in a small group of cardiac EST cases revealed a marked ΔIGFBP-3 between 0 and 150 min in positive test cases, that was absent in negative normal controls. Further assessment in EST cases in more heterogeneous ED patients also found significant (albeit muted) 0–2 h positive ΔIGFBP-3 in inducible ischaemic cases. It is important to note here that the direction of ΔIGFBP-3 in EST cases is negative, whereas in ED cases, it was positive. This is mostly likely due to the timing of sampling, such that ED cases have their first sample taken some hours post-symptom onset—when IGFBP-3 has reached/is close to the nadir seen at 120–150 min in the experimental EST (*Figure 2*)—with the second at 2 h reflecting the rise which was seen with later samples in EST. Proper assessment of the ability of ΔIGFBP-3 to improve the diagnostic yield from cardiac EST will require more rigorous assessment in an appropriately designed and recruited study population with sampling at the time of testing.

In contrast with cardiac EST, there was no discernible ΔIGFBP-3 in patients undergoing the non-ischaemic toxic challenge of SAA, out to 24 h post-procedure. A similar result was observed in ED patients suffering acute MI (ROC AUC = 0.52) in the ED cohorts that followed. This suggests that plasma IGFBP-3 may be less responsive to immune system/inflammatory/free radical processes terminal necrotic events such as SAA/MI, possibly due to inhibition of protease activity relevant to IGFBP-3 metabolism, but this requires further studies for confirmation.

Regional arterio-venous assessment of IGFBP-3 indicated net cardiac and hepatic extraction/degradation of IGFBP-3. To our knowledge, our data is the first to show both potential hepatic and cardiac extraction of IGFBP-3 in humans and in this regard, IGFBP-3 has a similar human profile to that of IGFBP-7.^25^ Reconciliation of the finding of cardiac extraction of IGFBP-3 in humans with our discovery of IGFBP-3 in IIRH perfusates could come from consideration of the IIRH as an isolated organ and the fact that tissue expression of IGFBP-3 is higher in other organs compared with the heart.^36,37^ The validity of our arterio-venous results is supported by complementary regional hs-TnT data which showed the expected cardiac release and renal clearance, as we have published previously.^17^

In ED patients from three well-published and similar studies, IGFBP-3 did not detect acute MI, but it did improve on the yield of patients satisfying the NOT rule. Further, among patients eligible for NOT rule inclusion, ΔIGFBP-3 provided potential discrimination with respect to three important considerations: the presence of UAP, 70% arterial stenosis on imaging, and whether benefit might accrue from further stress testing. Despite concomitant advances in troponin testing/clinical pathways to improve the identification of high- and low-risk ED cases, a sizeable fraction (25–30%) still have clinically uncertain presentations and identification of those who require further objective testing (either physical or imaging) would benefit from additional biomarker support. Graded troponin levels below mandated cut-offs have been proposed to assist with the identification of significant CAD and subsequent events,^8,9^ but do not assist in the detection of UAP. The PPV increments provided for UAP by IGFBP-3 are modest, but for the prediction of 70% stenosis in sub-threshold hs-TnT cases, IGFBP-3 + ΔIGFBP-3 provided a reasonable shift of 9% of intermediate risk cases—primarily to low risk—with no false negatives; if validated by further studies, this latter improvement could be of important clinical benefit to optimize angiography requests. Non-cardiac inflammatory influences on the observed responses of IGFBP-3 cannot be ruled out, but it is noteworthy that in the identification of sole infective disease in our ED cohorts, IGFBP-3 levels did not change between 0 and 2 h sampling times. Also, of interest, the potential for the performance of IGFBP-3 to be enhanced by other factors involved in the IGF-1/IGFBP-3 binding complex remains to be determined, including the determination of 70% stenosis outcome prediction at 365 days, where IGFBP-3 was superior to hs-TnT.

The precise biochemical mechanisms underpinning the performance of IGFBP-3 + ΔIGFBP-3 in chest pain/ACS patients are likely to involve multiple known proteases and plasma binding factors,^35^ all with cardiovascular localization and relevance. Firstly, IGFBP-3 RNA and protein, as well as IGF-1, are present in smooth muscle cells (SMCs) from atherosclerotic plaques taken from patients who experienced crescendo angina, but not in SMC from normal coronary artery specimens;^38^ secondly, in a porcine model of embolization induced ischaemia, cardiac myocytes produced elevated levels of IGFBP-3 mRNA at 3–24 h post-onset;^39^ further, IGFBP-3 RNA and protein are both expressed in human ventricles and are more abundant in failing hearts from patients with IHD compared with HCM.^40^ Thirdly, IGFBP-3 promotes angiogenesis in human endothelial cells via IGF-I receptor signalling and sphingosine kinase and sphingosine-1-phospate formation,^41^ and the act of IGFBP-3 binding to endothelial cells inhibits its own proteolysis.^42^ A limited number of studies have addressed plasma measurement of IGFBP-3 as a prognostic marker of future cardiac ischaemic events,^43,44^ but in both cases, IGFBP-3 was predictive of future events independent of known clinical and biomarker risk variables. Thus, clear directions are present for studies addressing the mechanistic actions underlying our observations and the potential to enhance/optimize performance further.

### Study limitations

First, this report presents observational studies only and we cannot make inferences about the impact on patient outcomes. Second, despite all three ED studies employing guideline definitions for the diagnosis of UAP, we cannot exclude regional differences being present and influencing analyses. Further, we used the presence of 70% stenosis to define significant CAD. We did not perform more detailed analyses on plaque burden and vulnerability. Third, the adjudication of MI across all three studies utilized two different troponin assays. While our analysis used the FAST-TRAC study with Beckman hs-TnI assay for the definition of MI, both SPACE and APACE have been adjudicated with reference to Roche hs-cTnT. To limit the potential effects of this, we performed hs-cTnT measurements in FAST-TRAC to generate biochemical equivalence across all three studies with respect to analyses. Fourth, we cannot rule out statistical over-fitting in some analyses due to smaller case numbers and in that regard, some of the results are hypothesis-generating only. Further studies in appropriate patient groups with adequate sample size and event rates are required to validate the findings of this study.

## Conclusions

We provide the first translational report—from basic discovery to clinical assessment—that circulating IGFBP-3 is responsive to acute cardiac ischaemia (without necrosis) and that ΔIGFBP-3 has the potential to aid decision-making with regard to the need for imaging to detect significant coronary stenosis or the need to submit individuals for cardiac stress testing. This applies both to patients eligible for clinical scoring (e.g. NOT rule) and/or those with detectable troponin that does not reach actionable levels with respect to the diagnosis of ACS.

## Supplementary Material

oeaf028_Supplementary_Data

## Data Availability

The non-identifying data underlying this article will be shared on reasonable request to the corresponding author.

## Lead author biography

Chris J. Pemberton is a Research Professor based at the Christchurch Heart Institute, University of Otago, Christchurch, New Zealand, and a co-founder/VP Discovery at Upstream Medical Technologies. His focus is on the identification of new markers and therapies that meet unmet need in cardiac ischaemia and related diseases.

## References

[1] Byrne RA, Rossello X, Coughlan JJ, Barbato E, Berry C, Chieffo A, Claeys MJ, Dan GA, Dweck MR, Galbraith M, Gilard M, Hinterbuchner L, Jankowska EA, Jüni P, Kimura T, Kunadian V, Leosdottir M, Lorusso R, Pedretti RFE, Rigopoulos AG, Rubini Gimenez M, Thiele H, Vranckx P, Wassmann S, Wenger NK, Ibanez B; ESC Scientific Document Group. 2023 ESC guidelines for the management of acute coronary syndromes. Eur Heart J 2023;44:3720–3826. 37622654 10.1093/eurheartj/ehad191

[2] Twerenbold R, Costabel JP, Nestelberger T, Campos R, Wussler D, Arbucci R, Cortes M, Boeddinghaus J, Baumgartner B, Nickel CH, Bingisser R, Badertscher P, Puelacher C, du Fay de Lavallaz J, Wildi K, Rubini Giménez M, Walter J, Meier M, Hafner B, Lopez Ayala P, Lohrmann J, Troester V, Koechlin L, Zimmermann T, Gualandro DM, Reichlin T, Lambardi F, Resi S, Alves de Lima A, Trivi M, Mueller C. Outcome of applying the ESC 0/1-hour algorithm in patients with suspected myocardial infarction. J Am Coll Cardiol 2019;74:483–494. 31345421 10.1016/j.jacc.2019.05.046

[3] Chiang CH, Chiang CH, Pickering JW, Stoyanov KM, Chew DP, Neumann JT, Ojeda F, Sörensen NA, Su KY, Kavsak P, Worster A, Inoue K, Johannessen TR, Atar D, Amann M, Hochholzer W, Mokhtari A, Ekelund U, Twerenbold R, Mueller C, Bahrmann P, Buttinger N, Dooley M, Ruangsomboon O, Nowak RM, DeFilippi CR, Peacock WF, Neilan TG, Liu MA, Hsu WT, Lee GH, Tang PU, Ma KS, Westermann D, Blankenberg S, Giannitsis E, Than MP, Lee CC. Performance of the European Society of Cardiology 0/1-hour, 0/2-hour, and 0/3-hour algorithms for rapid triage of acute myocardial infarction: an international collaborative meta-analysis. Ann Intern Med 2022;175:101–113. 34807719 10.7326/M21-1499

[4] Gulati M, Levy PD, Mukherjee D, Amsterdam E, Bhatt DL, Birtcher KK, Blankstein R, Boyd J, Bullock-Palmer RP, Conejo T, Diercks DB, Gentile F, Greenwood JP, Hess EP, Hollenberg SM, Jaber WA, Jneid H, Joglar JA, Morrow DA, O'Connor RE, Ross MA, Shaw LJ. 2021 AHA/ACC/ASE/CHEST/SAEM/SCCT/SCMR guideline for the evaluation and diagnosis of chest pain: a report of the American College of Cardiology/American Heart Association Joint Committee on Clinical Practice Guidelines. Circulation 2021;144:e368–e454. 10.1161/CIR.0000000000001029 34709879

[5] Greenslade JH, Parsonage W, Than M, Scott A, Aldous S, Pickering JW, Hammett CJ, Cullen L. A clinical decision rule to identify emergency department patients at low risk for acute coronary syndrome who do not need objective coronary artery disease testing: the no objective testing rule. Ann Emerg Med 2016;67:478–489.e2. 26363570 10.1016/j.annemergmed.2015.08.006

[6] Ratmann PD, Boeddinghaus J, Nestelberger T, Lopez-Ayala P, Koechlin L, Wildi K, Miro O, Martín-Sánchez FJ, Christ M, Twerenbold R, Rubini Gimenez M, Keller DI, Mueller C; Advantageous Predictors of Acute Coronary Syndrome Evaluation Investigators. External validation of the no objective testing rules in acute chest pain. J Am Heart Assoc 2021;10:e020031. 33977760 10.1161/JAHA.120.020031 PMC8200689

[7] Ratmann PD, Boeddinghaus J, Nestelberger T, Lopez-Ayala P, Huré G, Gehrke J, Koechlin L, Wildi K, Mueller P, Bima P, Wussler D, Gisler N, Miro O, Martín-Sánchez FJ, Christ M, Gualandro DM, Twerenbold R, Gimenez MR, Keller DI, Buser A, Mueller C; APACE Investigators. Extending the no objective testing rules to patients triaged by the European Society of Cardiology 0/1-hour algorithms. Eur Heart J Acute Cardiovasc Care 2022;11:834–840. 36179255 10.1093/ehjacc/zuac120

[8] Than MP, Aldous SJ, Troughton RW, Pemberton CJ, Richards AM, Frampton CMA, Florkowski CM, George PM, Bailey S, Young JM, Cullen L, Greenslade JH, Parsonage WA, Everett BM, Peacock WF, Jaffe AS, Pickering JW. Detectable high-sensitivity cardiac troponin within the population reference interval conveys high 5-year cardiovascular risk: an observational study. Clin Chem 2018;64:1044–1053. 29760219 10.1373/clinchem.2017.285700

[9] Lee KK, Bularga A, O'Brien R, Ferry AV, Doudesis D, Fujisawa T, Kelly S, Stewart S, Wereski R, Cranley D, van Beek EJR, Lowe DJ, Newby DE, Williams MC, Gray AJ, Mills NL. Troponin-guided coronary computed tomographic angiography after exclusion of myocardial infarction. J Am Coll Cardiol 2021;78:1407–1417. 34593122 10.1016/j.jacc.2021.07.055 PMC8482793

[10] Ho AFW, Yau CE, Ho JS, Lim SH, Ibrahim I, Kuan WS, Ooi SBS, Chan MY, Sia CH, Mosterd A, Gijsberts CM, de Hoog VC, Bank IEM, Doevendans PA, de Kleijn DPV. Predictors of major adverse cardiac events among patients with chest pain and low HEART score in the emergency department. Int J Cardiol 2024;395:131573. 37931658 10.1016/j.ijcard.2023.131573

[11] Meier M, Boeddinghaus J, Nestelberger T, Koechlin L, Lopez-Ayala P, Wussler D, Walter JE, Zimmermann T, Badertscher P, Wildi K, Giménez MR, Puelacher C, Glarner N, Magni J, Miró Ò, Martin-Sanchez FJ, Kawecki D, Keller DI, Gualandro DM, Twerenbold R, Nickel CH, Bingisser R, Mueller C; APACE Investigators. Comparing the utility of clinical risk scores and integrated clinical judgement in patients with suspected acute coronary syndrome. Eur Heart J Acute Cardiovasc Care 2023;12:693–702. 37435949 10.1093/ehjacc/zuad081 PMC10599640

[12] Mbikou P, Rademaker MT, Charles CJ, Richards AM, Pemberton CJ. Cardiac effects of DWORF (dwarf open reading frame) peptide in normal and ischaemia/reperfused isolated rat hearts. Peptides 2020;124:170192. 31712056 10.1016/j.peptides.2019.170192

[13] Appleby S, Aitken-Buck HM, Holdaway MS, Byers MS, Frampton CM, Paton LN, Richards AM, Lamberts RR, Pemberton CJ. Cardiac effects of myoregulin in ischemia-reperfusion. Peptides 2024;174:171156. 38246425 10.1016/j.peptides.2024.171156

[14] Bioanalytical Method Validation: Guidance for Industry . U.S. Department of Health and Human Services, Food and Drug Administration, Center for Drug Evaluation and Research (CDER), Center for Veterinary Medicine (CVM), May 2018, Biopharmaceutics. Accessed 20 August, 2019. https://www.fda.gov/files/drugs/published/Bioanalytical-Method-Validation-Guidance-for-Industry.pdf.

[15] Liebetrau C, Gaede L, Dörr O, Blumenstein J, Rosenburg S, Hoffmann J, Troidl C, Hamm CW, Nef HM, Möllmann H, Richards AM, Pemberton CJ. Reference values and release kinetics of B-type natriuretic peptide signal peptide in patients with acute myocardial infarction. Clin Chem 2015;61:1532–1539. 26506995 10.1373/clinchem.2015.244327

[16] Liebetrau C, Gaede L, Wolter JS, Homann J, Meyer A, Dörr O, Nef HM, Troidl C, Hamm CW, Möllmann H, Richards AM, Pemberton CJ. Release kinetics of high-sensitivity cardiac troponins I and T and troponin T upstream open reading frame peptide (TnTuORF) in clinically induced acute myocardial infarction. Biomarkers 2017;22:304–310. 27775442 10.1080/1354750X.2016.1252965

[17] Lee J, Young J, Frampton CM, Aldous S, Troughton RW, Than M, Richards AM, Pemberton CJ. A novel troponin T peptide in humans: assay, biochemistry and preliminary findings in acute coronary syndromes. Int J Cardiol 2015;190:68–74. 25918054 10.1016/j.ijcard.2015.04.145

[18] Chew-Harris J, Appleby S, Richards AM, Troughton R, Pemberton CJ. Analytical, biochemical and clearance considerations of soluble urokinase plasminogen activator receptor (suPAR) in healthy individuals. Clin Biochem 2019;69:36–44. 31129182 10.1016/j.clinbiochem.2019.05.010

[19] Advantageous Predictors of Acute Coronary Syndromes Evaluation (APACE) Study . Clinical trials.gov identifier: NCT00470587. https://classic.clinicaltrials.gov/ct2/show/NCT00470587 (accessed 24 January 2024).

[20] Peacock WF, Maisel AS, Mueller C, Anker SD, Apple FS, Christenson RH, Collinson P, Daniels LB, Diercks DB, Somma SD, Filippatos G, Headden G, Hiestand B, Hollander JE, Kaski JC, Kosowsky JM, Nagurney JT, Nowak RM, Schreiber D, Vilke GM, Wayne MA, Than M. Finding acute coronary syndrome with serial troponin testing for rapid assessment of cardiac ischemic symptoms (FAST-TRAC): a study protocol. Clin Exp Emerg Med 2022;9:140–145. 35843615 10.15441/ceem.21.154 PMC9288884

[21] Pickering JW, Young JM, George PM, Watson AS, Aldous SJ, Troughton RW, Pemberton CJ, Richards AM, Cullen LA, Than MP. Validity of a novel point-of-care troponin assay for single-test rule-out of acute myocardial infarction. JAMA Cardiol 2018;3:1108–1112. 30347004 10.1001/jamacardio.2018.3368 PMC6583693

[22] Pickering JW, Than MP, Cullen L, Aldous S, Ter Avest E, Body R, Carlton EW, Collinson P, Dupuy AM, Ekelund U, Eggers KM, Florkowski CM, Freund Y, George P, Goodacre S, Greenslade JH, Jaffe AS, Lord SJ, Mokhtari A, Mueller C, Munro A, Mustapha S, Parsonage W, Peacock WF, Pemberton C, Richards AM, Sanchis J, Staub LP, Troughton R, Twerenbold R, Wildi K, Young J. Rapid rule-out of acute myocardial infarction with a single high-sensitivity cardiac troponin T measurement below the limit of detection: a collaborative meta-analysis. Ann Intern Med 2017;166:715–724. 28418520 10.7326/M16-2562

[23] Aldous S, Richards AM, Cullen L, Pickering JW, Than M. The incremental value of stress testing in patients with acute chest pain beyond serial cardiac troponin testing. Emerg Med J 2016;33:319–324. 26511125 10.1136/emermed-2015-204823

[24] Authors/Task Force Members; Elliott PM, Anastasakis A, Borger MA, Borggrefe M, Cecchi F, Charron P, Hagege AA, Lafont A, Limongelli G, Mahrholdt H, McKenna WJ, Mogensen J, Nihoyannopoulos P, Nistri S, Pieper PG, Pieske B, Rapezzi C, Rutten FH, Tillmanns C, Watkins H. 2014 ESC guidelines on diagnosis and management of hypertrophic cardiomyopathy: the task force for the diagnosis and management of hypertrophic cardiomyopathy of the European Society of Cardiology (ESC). Eur Heart J 2014;35:2733–2779. 25173338 10.1093/eurheartj/ehu284

[25] Tan ESJ, Chan SP, Choi YC, Pemberton CJ, Troughton R, Poppe K, Lund M, Devlin G, Doughty RN, Richards AM. Regional handling and prognostic performance of circulating insulin-like growth factor binding protein-7 in heart failure. JACC Heart Fail 2023;11:662–674. 37286261 10.1016/j.jchf.2023.01.016

[26] Appleby S, Chew-Harris J, Troughton RW, Richards AM, Pemberton CJ. Analytical and biological assessment of circulating human erythroferrone. Clin Biochem 2020;79:41–47. 32032568 10.1016/j.clinbiochem.2020.02.001

[27] Thygesen K, Alpert JS, Jaffe AS, Chaitman BR, Bax JJ, Morrow DA, White HD; Executive Group on behalf of the Joint European Society of Cardiology (ESC)/American College of Cardiology (ACC)/American Heart Association (AHA)/World Heart Federation (WHF) Task Force for the Universal Definition of Myocardial Infarction. Fourth universal definition of myocardial infarction (2018). J Am Coll Cardiol 2018;72:2231–2264. 30153967 10.1016/j.jacc.2018.08.1038

[28] Puelacher C, Gugala M, Adamson PD, Shah A, Chapman AR, Anand A, Sabti Z, Boeddinghaus J, Nestelberger T, Twerenbold R, Wildi K, Badertscher P, Rubini Gimenez M, Shrestha S, Sazgary L, Mueller D, Schumacher L, Kozhuharov N, Flores D, du Fay de Lavallaz J, Miro O, Martín-Sánchez FJ, Morawiec B, Fahrni G, Osswald S, Reichlin T, Mills NL, Mueller C. Incidence and outcomes of unstable angina compared with non-ST-elevation myocardial infarction. Heart 2019;105:1423–1431. 31018955 10.1136/heartjnl-2018-314305

[29] Chin E, Zhou J, Dai J, Baxter RC, Bondy CA. Cellular localization and regulation of gene expression for components of the insulin-like growth factor ternary binding protein complex. Endocrinology 1994;134:2498–2504. 7515002 10.1210/endo.134.6.7515002

[30] Scharf JG, Schmidt-Sandte W, Pahernik SA, Koebe HG, Hartmann H. Synthesis of insulin-like growth factor binding proteins and of the acid-labile subunit of the insulin-like growth factor ternary binding protein complex in primary cultures of human hepatocytes. J Hepatol 1995;23:424–430. 8655960 10.1016/0168-8278(95)80201-0

[31] Koomen JM, Wilson CR, Guthrie P, Androlewicz MJ, Kobayashi R, Taegtmeyer H. Proteomone analysis of isolated perfused organ effluent as a novel model for protein biomarker discovery. J Proteome Res 2006;5:177–182. 16396509 10.1021/pr050170g

[32] Cordwell SJ, Edwards AV, Liddy KA, Moshkanbaryans L, Solis N, Parker BL, Yong AS, Wong C, Kritharides L, Hambly BD, White MY. Release of tissue-specific proteins into coronary perfusate as a model for biomarker discovery in myocardial ischemia/reperfusion injury. J Proteome Res 2012;11:2114–2126. 22250753 10.1021/pr2006928

[33] Ibrahim NE, Afilalo M, Chen-Tournoux A, Christenson RH, Gaggin HK, Hollander JE, Kastner P, Levy PD, Mang A, Masson S, Nagurney JT, Nowak RM, Pang PS, Peacock WF, Dipl-Stat VR, Walters EL, Januzzi JL Jr. Diagnostic and prognostic utilities of insulin-like growth factor binding protein-7 in patients with dyspnea. JACC Heart Fail 2020;8:415–422. 32354416 10.1016/j.jchf.2020.02.009

[34] Renes JS, van Doorn J, Hokken-Koelega ACS. Current insights into the role of the growth hormone-insulin-like growth factor system in short children born small for gestational age. Horm Res Paediatr 2019;92:15–27. 31509834 10.1159/000502739 PMC6979433

[35] Varma Shrivastav S, Bhardwaj A, Pathak KA, Shrivastav A. Insulin-like growth factor binding protein-3 (IGFBP-3): unraveling the role in mediating IGF-independent effects within the cell. Front Cell Dev Biol 2020;8:286. 32478064 10.3389/fcell.2020.00286 PMC7232603

[36] Arany E, Zabel P, Hill DJ. Rapid clearance of human insulin-like growth factor binding protein-3 from the rat circulation and cellular localization in liver, kidney and stomach. Growth Regul 1996;6:32–41. 8717448

[37] Boes M, Dake BL, Booth BA, Sandra A, Bateman M, Knudtson KL, Bar RS. IGF-I and IGFBP-3 transport in the rat heart. Am J Physiol Endocrinol Metab 2003;284:E237–E239. 12485812 10.1152/ajpendo.00336.2002

[38] Grant MB, Wargovich TJ, Ellis EA, Tarnuzzer R, Caballero S, Estes K, Rossing M, Spoerri PE, Pepine C. Expression of IGF-I, IGF-I receptor and IGF binding proteins-1, -2, -3, -4 and -5 in human atherectomy specimens. Regul Pept 1996;67:137–144. 8988513 10.1016/s0167-0115(96)00124-3

[39] Kluge A, Zimmermann R, Weihrauch D, Mohri M, Sack S, Schaper J, Schaper W. Coordinate expression of the insulin-like growth factor system after microembolisation in porcine heart. Cardiovasc Res 1997;33:324–331. 9074696 10.1016/s0008-6363(96)00236-2

[40] Granata R, Broglio F, Migliorino D, Cutrupi S, Baldanzi G, Sireno M, Fubini A, Grazian A, Ghigo E, Pucci A. Neonatal and adult human heart tissues from normal subjects and patients with ischemic, dilated or hypertrophic cardiomyopathy express insulin-like growth factor binding protein-3 (IGFBP-3). J Endocrinol Invest 2000;23:724–726. 11194704 10.1007/BF03345060

[41] Granata R, Trovato L, Lupia E, Sala G, Settanni F, Camussi G, Ghidoni R, Ghigo E. Insulin-like growth factor binding protein-3 induces angiogenesis through IGF-I- and SphK1-dependent mechanisms. J Thromb Haemost 2007;5:835–845. 17388800 10.1111/j.1538-7836.2007.02431.x

[42] Booth BA, Boes M, Dake BL, Knudtson KL, Bar RS. IGFBP-3 binding to endothelial cells inhibits plasmin and thrombin proteolysis. Am J Physiol Endocrinol Metab 2002;282:E52–E58. 11739083 10.1152/ajpendo.2002.282.1.E52

[43] Fischer F, Schulte H, Mohan S, Tataru M-C, Köhler E, Assmann G, von Eckardstein A. Associations of insulin-like growth factors, insulin-like growth factor binding proteins and acid-labile subunit with coronary heart disease. Clin Endocrinol 2004;61:595–602. 10.1111/j.1365-2265.2004.02136.x 15521962

[44] Kaplan RC, McGinn AP, Pollak MN, Kuller LH, Strickler HD, Rohan TE, Cappola AR, Xue X, Psaty BM. Association of total insulin-like growth factor-I, insulin-like growth factor binding protein-1 (IGFBP-1), and IGFBP-3 levels with incident coronary events and ischemic stroke. J Clin Endocrinol Metab 2007;92:1319–1325. 17264182 10.1210/jc.2006-1631

