# Supplementary material for: Insulin-like growth factor binding protein-3 (IGFBP-3): a biomarker of coronary artery disease induced myocardial ischaemia
Source: Eur Heart J Open. 2025 Mar 20;5(2):oeaf028. doi: 10.1093/ehjopen/oeaf028 (PMC11961406; doi:10.1093/ehjopen/oeaf028)
Supplement: oeaf028_Supplementary_Data [file oeaf028_supplementary_data.zip › IGFBP3 paper 2024 Supplementary Methods R2 MARCH.docx]

**Supplemental Methods**

**Insulin-like Growth Factor Binding Protein-3 (IGFBP-3): a biomarker of coronary artery disease (CAD) induced myocardial ischemia**

Lee et al.

**Isolated perfused heart protocol**

Ischemia was induced by ligation in isolated rat hearts prepared via the Langendorff isolated rat heart method. Sprague Dawley (mol) rats (all male) were anaesthetised with sodium pentobarbital (60 mg/Kg, intraperitoneal) and the heart rapidly excised, placed into ice-cold perfusion buffer and mounted on the Langendorff apparatus. The aorta was cannulated above the aortic valve, perfused with a peristaltic pump (MP-2, Gilson Minipuls) at 12 mL/min (constant retrograde flow) with a Krebs-Henseleit solution (122.8 mM NaCl, 22 mM NaHCO_3_, 1.2 mM KH_2_PO_4_, 1.1 mM MgSO_4_.7H_2_O, 4.7 mM KCl, 11 mM glucose) containing 1.5 mM calcium, gassed with 95% O_2_ / 5% CO_2_ (pH 7.40), paced at 310bpm (5.2 Hz) and maintained at 37ºC. Left ventricular hemodynamic contractile parameters were measured by inserting a custom-made polyvinyl chloride balloon (attached to a pressure transducer, via fluid filled tubing, MLT-844/Advance Digital Instruments, ADInstruments) into the left ventricle (LV). The balloon was inflated to yield an LV end-diastolic pressure (LVEDP) of 10 − 15 mmHg at the beginning of the stabilization period and not adjusted thereafter. LV pressure was digitally processed to yield systolic developed pressure (DP), LVEDP, heart rate and the maximum and minimum derivatives of LV pressure (dP/dt(max) and dP/dt(min), respectively). A side-arm cannula attached to a second pressure transducer was inserted into the aortic cannula above the heart to measure perfusion pressure (PP), an indirect measure of coronary arterial tone. Hearts were allowed to stabilise for 30-minutes before the experimental protocol commenced. Ischemia with continuing coronary flow was induced by reversible ligation using a 5-0 suture and a small 5mm length of PVC tube (n=3, experimental) around the left main coronary artery at a distance of ~20% from the apex of the heart; the PVC tube was compressed onto the artery by tightening a snare knot to induce complete closure. Control/sham hearts (n=3) received 5-0 suture without snare knot ligature. Constant flow (12ml/min) was maintained during application of experimental/sham protocol for 30 minutes after ligature. Perfusate samples (25mL volume) were collected at -10, 0, +10, +20 and +30 minutes relative to snare ligature for MS analyses. All haemodynamic data were recorded using a Powerlab data acquisition system coupled with Chart5 software (ADInstruments) continuously collected throughout the experiment.

**MS biomarker discovery protocol**

Collected control or ligated rat perfusate was mixed with ice cold acetonitrile (1:3 ratio of perfusate to acetonitrile) to precipitate protein. The proteins were tryptic digested after reduction with dithiothreitol (DTT) and alkylation with iodoacetamide (IAM). Solid phase extraction (C18) was performed to remove interfering matrix components. Samples were separated on a Jupiter Proteo® C12 reversed phase HPLC column (150 x 2 mm, 4 μm, 90 Ǻ, Phenomenex, CA, USA) using a Dionex UltiMate™ 3000 SD HPLC system (Thermo Scientific, MA, USA). An acetonitrile gradient from 95% A (0.1% formic acid in water) to 65% B (0.1% formic acid in acetonitrile) was run over 46 min at a flow rate of 200 µL/min. The column compartment was heated to 40°C and the autosampler was cooled to 5°C. The HPLC was coupled inline to a Heated ESI ionisation source of a Velos Pro Ion Trap mass spectrometer (Thermo Scientific, MA, USA). MS data were acquired in positive mode. The Heated ESI source settings were: 4 kV, nitrogen sheath gas 15 (arb), aux gas 5 (arb), capillary 275°C, heater 400°C. A Nth Order Double Play was performed. The first event was a full scan from 390 to 2000 m/z followed by CID MS/MS of the most intense ion for the top 6 ions detected in the full scan. No exclusion lists were used. Data analysis was performed with Proteome Discoverer 2.5.0.400 (Thermo Scientific, MA, USA) with the output forming the basis of perfusate discovery from the IPRH and MS protocol (Figure S1).

**
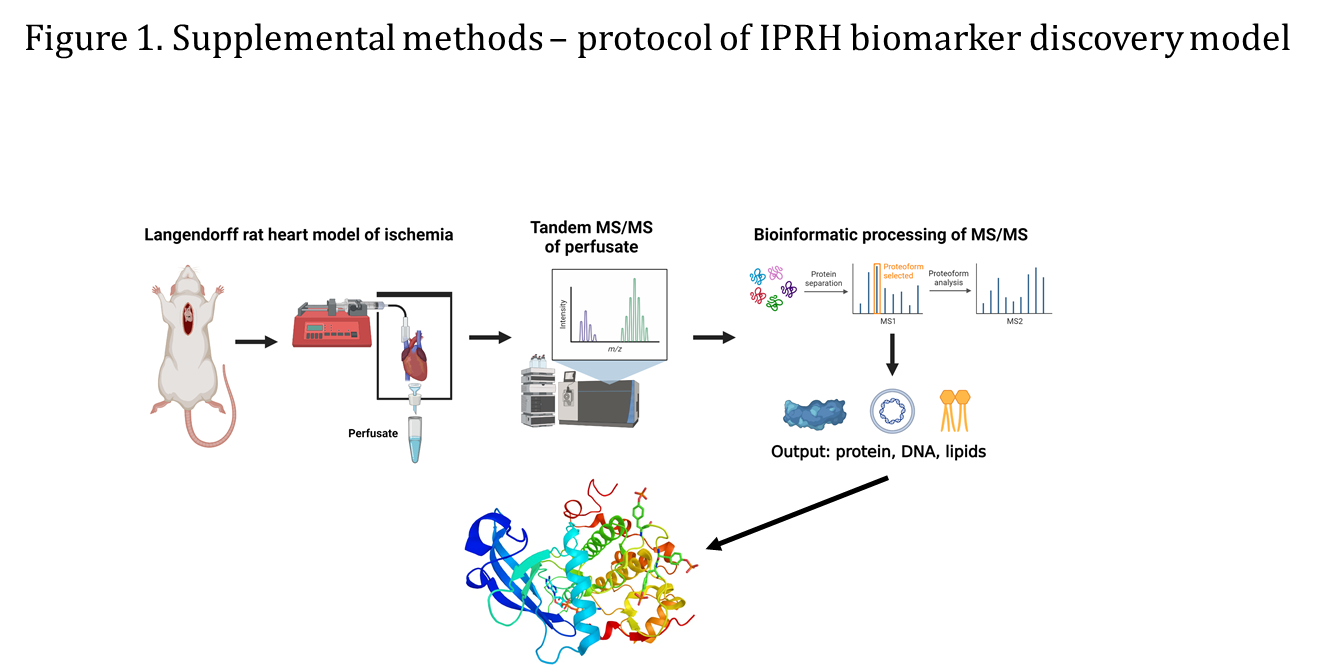
**

**Figure S1.** Schematic of the IPRH + MS biomarker discovery protocol.

**IGFBP-3 assay protocol and validation**

Human IGFBP-3 was measured on samples diluted 100x in assay buffer (0.1M PBS, 2% BSA) in a sandwich antibody ELISA. Plates (96-well) are pre-coated with a monoclonal antibody targeting amino acids 159-163 of recombinant human IGFBP-3. 100µl of samples and calibrators (0-50ng/mL) were incubated for 2 hours with 100µl of a binding enhancement buffer (proprietary) to reduce non-specific protein interactions. After washing, 200µl of HRP-conjugated polyclonal antibody that recognises a complex tertiary structure array of amino and carboxy terminal residues in human IGFBP-3 was added and mixed for a further 2hours. After final washing, plates were incubated for 20-30min with 200µl of a TMB substrate to develop colour. This reaction was stopped with 50µl of 2N sulfuric acid and plates read at 450nM with correction at 540nM. Low and high quality control (QC) samples were run at 6.5 and 23.9ng/mL respectively, across the main sample reading portion of the assay curve. Diluted sample values were adjusted for dilution after OD readings.

Cross-reactivities of this assay with the following markers are all <0.1%: cTnT, cTnI, cTnC, skeletal TnT, NT-proBNP, GDF-15, MR-proADM, Copeptin, IGF-1, IGF-II, IGFBP-1, IGFBP-2, IGFBPs 4-7, sFlt-3 and FGFs4-10.

*Effect of collection tube type upon endogenous immunoreactive IGFBP-3 in human blood* EDTA and Heparin plasma samples from 36 healthy volunteers were evaluated for measured levels of IGFBP-3. Median (IQR) levels of IGFBP-3 from the two sample types were not significantly different from each other (EDTA = 1931 (1661-2121) ng/mL versus Heparin 1923 (1770-2241) ng/mL, p=0.45). When assessed by regression, the achieved r^2^ = 0.86 and the mean ± SD ratio of EDTA/Heparin was 1.06±0.08. Further, individual matched samples gave very similar profiles when compared (supplementary Figure S2).


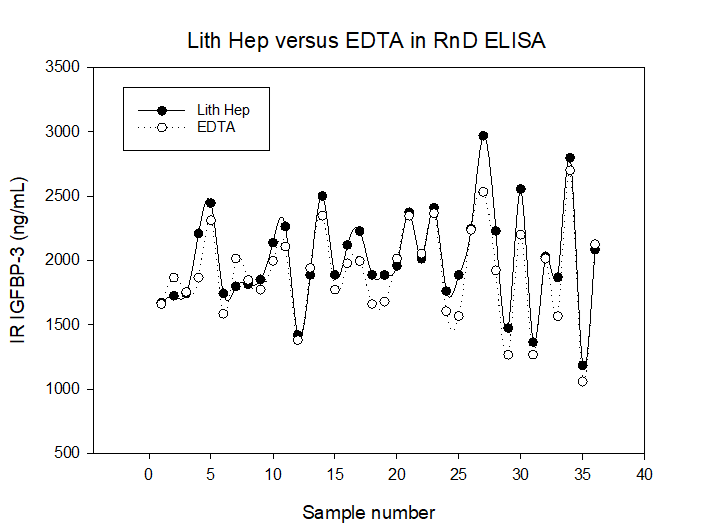


**Figure S2.** Matched samples assessed for sample type (LithHep versus EDTA).

*Effect of freeze thaw of plasma samples upon measured IGFBP-3 levels*

Eight randomly chosen EDTA plasma samples were aliquoted and underwent 3 freeze thaw cycles over 3 weeks (ie. 1 per week). IGFBP-3 was measured in each sample after thawing. This demonstrated IGFBP-3 measurement using the assay is acceptable up to 3 freeze thaws as levels decreased by ~10% on the third thaw.

*Sample interference testing*

Haemolysis at greater than 2.5g/L (tested range of 0.5-10g/L) and lipaemia at greater than 2g/L (tested range of 1-6g/L) both depressed IGFBP-3 values in EDTA plasma by >10% (n=4 samples for each assessment). Thus, samples that met either of these levels were not used for studies contained in this report.

*Western blot verification of assay detected IGFBP-3*

Plasma IGFBP-3 protein captured by the immunoassay monoclonal antibody was eluted from the ELISA plate using 0.1M glycine. Following neutralisation of the samples with 1 M Tris (pH 7.4-7.7) concentrated samples were separated on AnyKD precast gels (#4569033, Bio-Rad) using sodium dodecyl sulfate–polyacrylamide gel electrophoresis (SDS-PAGE) under denaturing conditions. The proteins were then transferred onto 0.2 µm polyvinylidene fluoride (PVDF) membrane (#10600101, Cytiva). The membranes were blocked using 5% (w/v) skim milk in Tris-buffered saline containing 0.1% Tween-20 (TBS-T) for 1 hour at room temperature. Following the blocking step, PVDF membranes were incubated overnight at 4°C with the detection polyclonal antibody conjugated to horseradish peroxidase (HRP). The membranes were then washed three times in TBST before being reacted with ECL detection reagent (#RPN2235, Cytiva) and imaged on Uvitec Gel Doc.

**Human cardiac stress testing protocol**

12 lead ECGs were obtained during each stage of the stress protocol, with additional 12 lead ECGs obtained at the end of the stress protocol and during recovery. Blood pressure was recorded 2 minutes into each stage of the stress protocol. EDTA blood samples were collected at baseline (just prior to the stress test) and then at 30, 60, 90, 120, 150, 180, 210, 240 minutes after the start of the stress test. Comprehensive transthoracic echocardiography (TTE) was performed pre- and post-stress testing using an iE33 ultrasound machine (Philips, Koninklijke Philips Electronics N.V.) and Prosolv Software (Prosolv, Indianapolis, Indiana). Echocardiography was performed according to American Society of Echcardiography recommendations.^1^ Pre-stress imaging included assessment of pre-existing wall motion abnormalities, with overall systolic and diastolic function. Post-stress echo, completed within two minutes of maximal exercise, assessed development of new regional wall motion abnormalities only. Patients with a positive stress ECG, but normal peak wall motion, were considered to have a negative stress test outcome. All TTE images were interpreted by certified echocardiographers, blinded to biomarker results. Any decision for patients to withhold their usual anti-anginal medications (including beta blockers) on the day of the stress test was at the discretion of the treating physician.

Septal alcohol ablation (SAA) - SEARCH Study

Clinical history, physical examination, 12-lead electrocardiography, laboratory tests, echocardiography and coronary angiography were documented. The diagnosis of HOCM was made per ESC 2014 guidelines.^2^ All patients received analgesic and anxiolytic pre-treatment during SAA, which was performed with temporary septal branch stenosis for selective therapeutic injection of 96% ethanol. All SAA procedures were performed in a single session with a single septal branch stenosis. During the procedure, the mean (SD) volume of ethanol administered was 1.58 (0.48) mL. The median stenosis time was 16.0 min [interquartile range (IQR) 12.5-27.1 min]. Postprocedural management included monitoring in the intensive care unit for 48h. The pre- and postprocedural data documented the success of the procedure as a reduction in the mean intraventricular pressure gradient.

**ED studies**

*SPACE*

Patients with chest pain suspicious of acute coronary syndromes (ACS) were prospectively enrolled into our ongoing observational study known as Signal Peptides in Acute Coronary Events (SPACE, <http://www.anzctr.org.au,ACTRN12609000057280>). All patients were enrolled in accord with protocols approved by the Health and Disabilities Ethics Committee of the Ministry of Health, New Zealand. All participants gave informed consent before recruitment and all investigations conformed to the principles of the Declaration of Helsinki. Eligible patients aged 18 years or older with the primary complaint of acute chest pain clinically suspicious of ACS and ≤ 4 h from onset were recruited. More general/atypical symptoms (such as fatigue, nausea, vomiting, sweating and faintness) were not used as inclusion criteria. Patients with end stage renal disease on dialysis were excluded.

*FAST-TRAC*

All participating institutions obtained local ethics committee approval to participate, and all enrolled patients provided written informed consent. Inclusion criteria specified that patients were at least 18 years of age and presenting to an ED within 6 hours of symptoms consistent with ACS, defined as chest discomfort/pain, squeezing/fullness in the chest, pain radiating to left or both arms, jaw pain, pain in back/neck/stomach, shortness of breath, cold sweat, nausea/vomiting, or light-headedness. Patients were excluded if they were in acute distress requiring immediate lifesaving intervention, if they had cardiopulmonary resuscitation (defibrillation or cardioversion within 24 hours of presentation to the ED), could not provide informed consent, had a terminal illness and were not expected to survive 6 months, or had trauma likely to be the cause of their ACS symptoms (e.g., penetrating wounds).

Case report forms included baseline patient demographics, history, physical exam, ECG results, diagnostic and laboratory test results, with data handling guidelines that provide definitions and specifications on how to complete the case report form. All information recorded on the case report form was required to have verifiable source documentation. Physicians evaluated and documented the presence of MI, UA, cardiac ischemia, and noncardiac ACS-like symptoms. After informed consent was obtained, blood draws were obtained at presentation, and 1, 2, 3 to 4, and 6 to 12 hours later. All blood draw times were ±30 minutes from the target and could occur while in the ED or after hospitalization. All draws were required for each subject, except patients who were clinically ruled out for ACS. Those discharged before 6 hours only had serial draws obtained up to the time of discharge.

*APACE*

The Advantageous Predictors of Acute Coronary Syndrome Evaluation (APACE) study is an ongoing prospective international multi-centre study including 12 centres in 5 countries aimed at advancing early diagnosis of MI. Adult patients presenting to the ED with acute chest discomfort were enrolled. Each patient had to provide written informed consent to be included in the study. Patients with terminal kidney disease requiring regular dialysis and patients in cardiogenic shock were excluded. For this analysis, patients were also excluded if the final diagnosis was unclear after adjudication and if the measurement of cardiac troponin at ED presentation was missing. The study was carried out according to the principles of the Declaration of Helsinki and approved by the local ethics committees as well as the Ethics Commission of Northwestern and Central Switzerland (EKNZ). Data were obtained and analysed according to the Strengthening the Reporting of Observational Studies in Epidemiology (STROBE) reporting guideline. All patients underwent clinical assessment, including standardized and detailed medical history with assessment of 34 predefined chest pain characteristics, vital signs, physical examination, 12-lead electrocardiography (ECG), continuous ECG rhythm monitoring, pulse oximetry, standard blood testing, and chest radiography, if indicated. Levels of cardiac troponins (cTn), including high-sensitivity cTn (hs-cTn) in some centres, were measured at presentation and serially thereafter if clinically indicated. Treatment of patients was left to the discretion of the attending physician.

1. Lang RM, Bierig M, Devereux RB, Flachskampf FA, Foster E, Pellikka PA, et al. Chamber Quantification Writing Group; American Society of Echocardiography's Guidelines and Standards Committee; European Association of Echocardiography. Recommendations for chamber quantification: a report from the American Society of Echocardiography's Guidelines and Standards Committee and the Chamber Quantification Writing Group, developed in conjunction with the European Association of Echocardiography, a branch of the European Society of Cardiology. *J Am Soc Echocardiogr* 2005;18:1440-63.
2. Authors/Task Force members; Elliott PM, Anastasakis A, Borger MA, Borggrefe M, Cecchi F, Charron P, et al. 2014 ESC Guidelines on diagnosis and management of hypertrophic cardiomyopathy: the Task Force for the Diagnosis and Management of Hypertrophic Cardiomyopathy of the European Society of Cardiology (ESC). *Eur Heart J* 2014;35:2733-79. <https://doi.org/10.1093/eurheartj/ehu284>
