# Supplementary material for: Insulin-like growth factor binding protein-3 (IGFBP-3): a biomarker of coronary artery disease induced myocardial ischaemia
Source: Eur Heart J Open. 2025 Mar 20;5(2):oeaf028. doi: 10.1093/ehjopen/oeaf028 (PMC11961406; doi:10.1093/ehjopen/oeaf028)
Supplement: oeaf028_Supplementary_Data [file oeaf028_supplementary_data.zip › IGFBP3 paper Supplementary Data R2 MARCH.docx]

**Supplemental Data**

**Insulin-like Growth Factor Binding Protein-3 (IGFBP-3): marker of sub-infarction coronary artery disease (CAD).**

Lee et al.

**Supplemental Tables**

**Table S1.** Demographics of SPICI study exercise stress testing (EST) cases. Median (IQR) or number (%). ***** = p<0.01

| **Characteristics** | **EST positive** | **EST negative** |
| --- | --- | --- |
| Patients, n | 6 | 6 |
| Age, yrs | 68 (65-74) | 39 (31-56) ***** |
| Sex, n *Male*  *Female* | 3 (50%)  3 (50%) | 3 (50%)  3 (50%) |
| **Comorbidities** |  |  |
| Known CAD | 4 (66) | 0 |
| Hypertension | 5 (83) | 0 |
| Cholesterol | 6 (100) | 0 |
| Diabetes | 0 | 0 |
| Smoking | 1 (17) | 0 |
| Family Hx of CVD | 5 (83) | 0 |
| Prior PCI | 3 (50) | 0 |
| **Medications** |  |  |
| Beta blocker | 6 (100) | 0 |
| Calcium blocker | 4 (66) | 0 |
| Nitrates | 4 (66) | 0 |
| Aspirin | 6 (100) | 0 |
| Statin | 6 (100) | 0 |
| **Test results** |  |  |
| ECG positive | 5 (83) | 0 |
| Positive symptoms | 6 (100) | 0 |
| Base LVEF | 58 (55-60) | 64 (61-68) |
| Echo positive | 6 (100) | 0 |
| Base heart rate | 68 (56-80) | 72 (67-75) |
| Peak heart rate | 135 (109-161) | 184 (167-198) ***** |
| Base SBP | 142 (128-156) | 120 (116-144) |
| Peak SBP | 196 (169-224) | 145 (113-176) |
| Base DBP | 83 (67-99) | 76 (68-84) |
| Peak DBP | 81 (64-98) | 69 (65-73) |
| Metabolic equivalents (METS) | 9.5 (5.5-13.1) | 16.5 (14.2-17.8) ***** |

**Table S2.** Demographics of regional sampling (GRADIENT) and SAA (SEARCH) patients. Median (IQR) or number (%). * = p<0.01. hsTnI was ABBOTT ARCHITECT.

| **Characteristics** | **GRADIENT** | **SEARCH** |
| --- | --- | --- |
| Patients, n | 14 | 14 |
| Age, yrs | 68 (64-71) | 65 (61-68) |
| Sex, n *Male*  *Female* | 12 (86)  2 (14) | 8 (57)  6 (43) |
| BMI (kg.m^2^) | 27 (26-30) | 28 (26-31) |
| **Comorbidities** |  |  |
| Known CAD | 4 (27) | 5 (34) |
| Hypertension | 8 (57) | 9 (64) |
| Cholesterol | 8 (57) | 3 (21) |
| Diabetes | 5 (36) | 1 (7) |
| Smoking | 6 (43) | 5 (36) |
| Family Hx of CVD | 4 (29) | 4 (29) |
| Prior CABG | 3 (21) | 0 (0) |
| NYHA *1*  *2*  *3*  *4* | 3 (21)  7 (50)  2 (14)  2 (14) | -  -  -  - |
| Hx of MI | 5 (36) | 0 |
| Hx of angina | 12 (86) | 5 (34) |
| Hx of heart failure | 3 (21) | 0 |
| **Medications** |  |  |
| Beta blocker | 12 (86) | - |
| Diuretics | 1 (7) | - |
| Nitrates | 1 (7) | - |
| Aspirin | 12 (86) | - |
| Statin | 10 (71) | - |
| ACEi/ARB | 9 (64) | - |
| **Hemodynamics** |  |  |
| E/E’ | 10 (8-14) | - |
| LVEF | 61 (54-64) | 66 (59-70) |
| Heart rate | 63 (60-66) | 67 (60-70) |
| SBP | 159 (143-163) | 156 (141-157) |
| DBP | 71 (66-77) | 72 (67-81) |
| **Clin. chemistry markers** |  |  |
| Na+, mmol/L | 138 (137-141) | 139 (138-141) |
| Hb, g/L | 141 (137-146) | 134 (119-139) |
| FA paO2, mmHg | 86 (79-99) | - |
| Peak hs-TnI, ng/L | 580 (210-1780) | 13650 (6868-20014) |
| GFR (ml/min/1.73m^2^) | 75 (65-79) | 80 (71-90) |

**Figure S1** – IGFBP-3 identification from ischemic isolated heart perfusate

1. Hemodynamics in isolated perfused rat hearts (n=3 per group) during 30 min ligation. Perfusate samples for MS/MS were taken at the earliest reversible ischemia time point of 10 min.
2. Base peak chromatogram of full scan masses of peptide retention times before (t=0) and during (t=10) ischemia induced by coronary artery ligation of rat hearts. The red and blue arrows indicate 2 novel unglycosylated IGFBP-3 derived peptides identified at their relative MS retention time (in minutes), with a high protein discovery score (FDR) confidence rating. IGFBP-3 protein was not present in the t=0 interrogation, nor was it present in a sham treated heart, suggesting it was induced early in the ischemia process. As a positive control cardiac troponin I was detected at 30 minutes after ligation induced ischemia (data not shown).

**
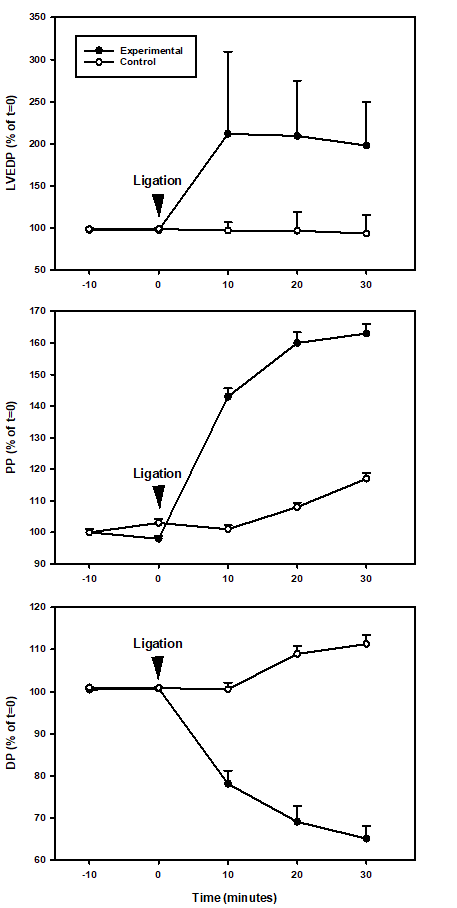
**

**A**

**
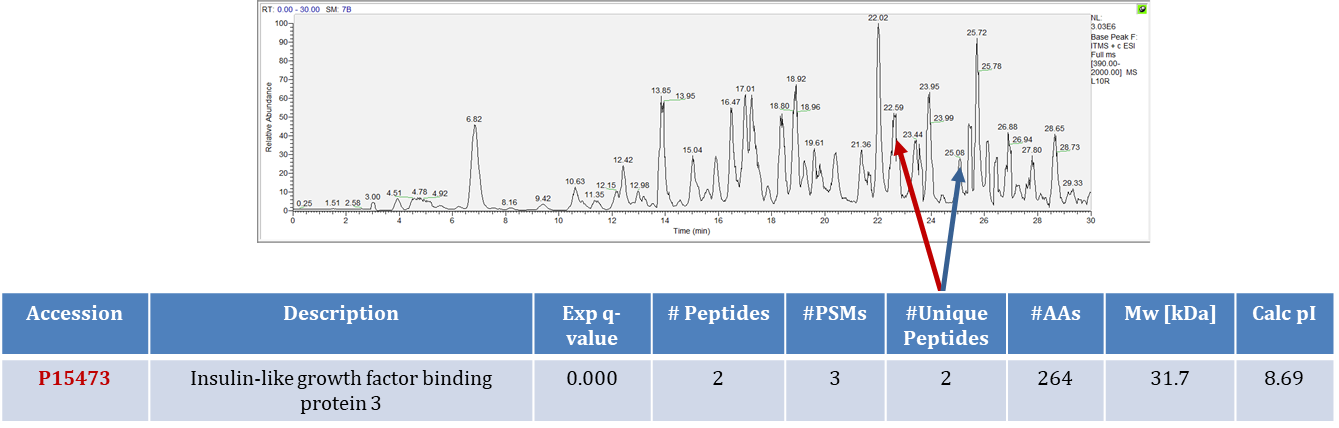
**

**B**

**Figure S2** – Western Blot analysis of IGFBP-3 forms measured in assay. Recombinant human IGFBP-3 (rhIGFBP3, lanes 1,2) and purified plasma protein (lanes 4,5) both eluted at Mr ~42-44kDa, consistent with glycosylated IGFBP-3 forms. Plasma contained an additional form at ~29kDa. Both IGFBP-3 sources had reduced Mr elution at ~37kDa after deglycosylation (lanes 3 and 6).


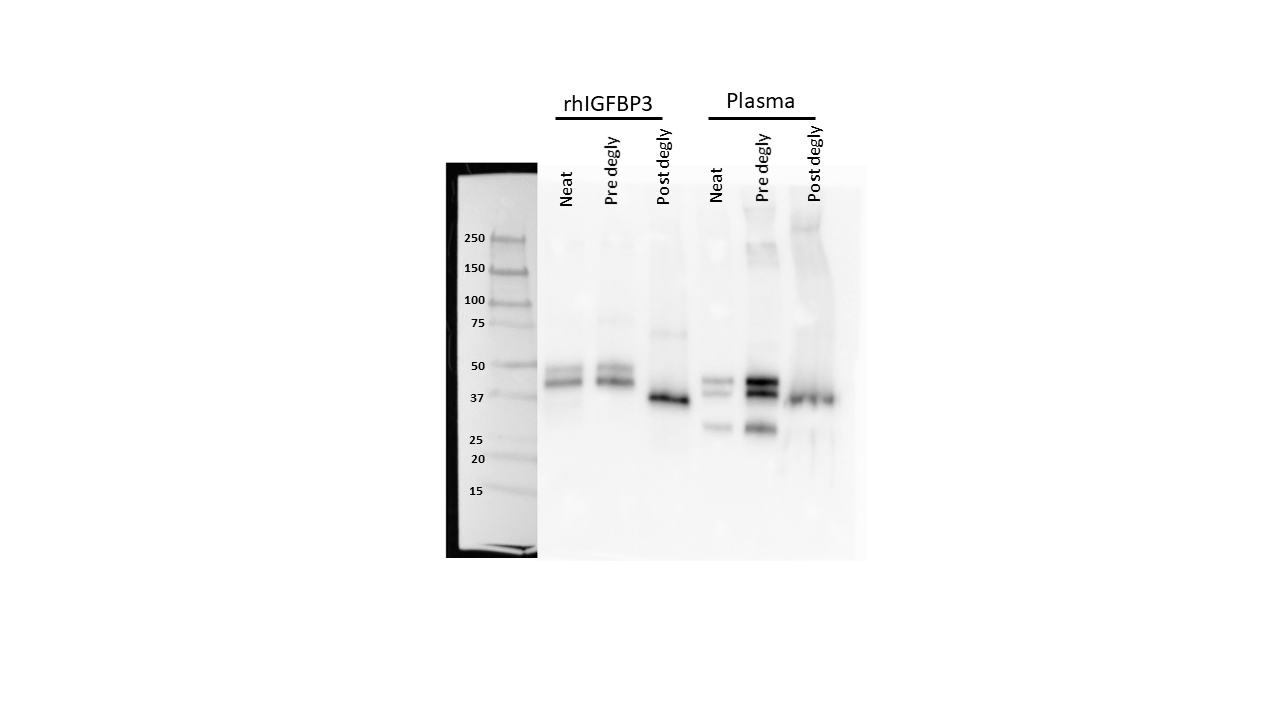


**Figure S3** – Final logistic regression models in SPSS for the diagnosis of UAP in (A) NOT rule eligible patients (n=1695 total, 123 UAP) and (B) all patients with maximum hsTnT between 5-14ng/L (n=1180 total, 119 UAP).

**A**


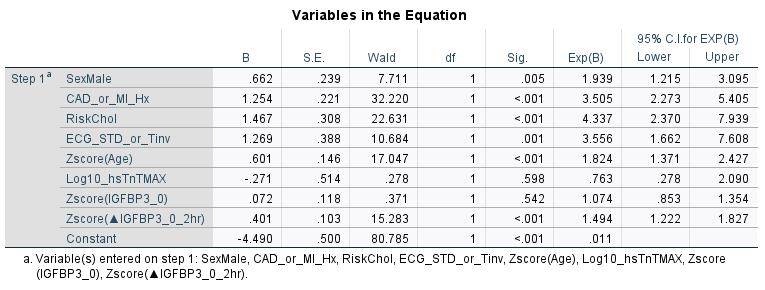


**B**


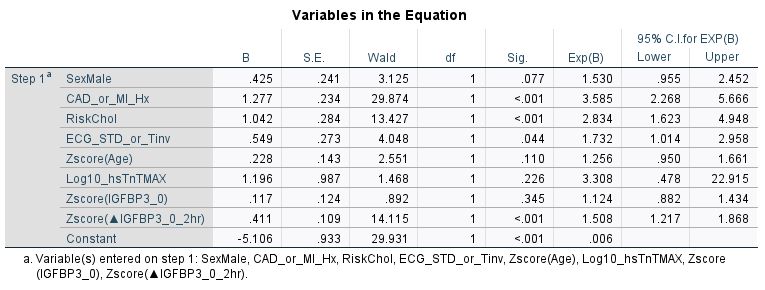


**Figure S4 -** Decision Curves corresponding to the Outcomes and Subgroups of Table 2.

**Baseline:** Clinical models as described in the text

**New:** Clinical model + IGFBP-3 + ∆IGFBP-3 (defined as t=2h minus the t=0h IGFBP-2 value) - where the new model (light blue line) is above the baseline (red) is the area of the probability space that the new model adds value

**Outcome:** UAP **Subgroup:** NOT rule eligible


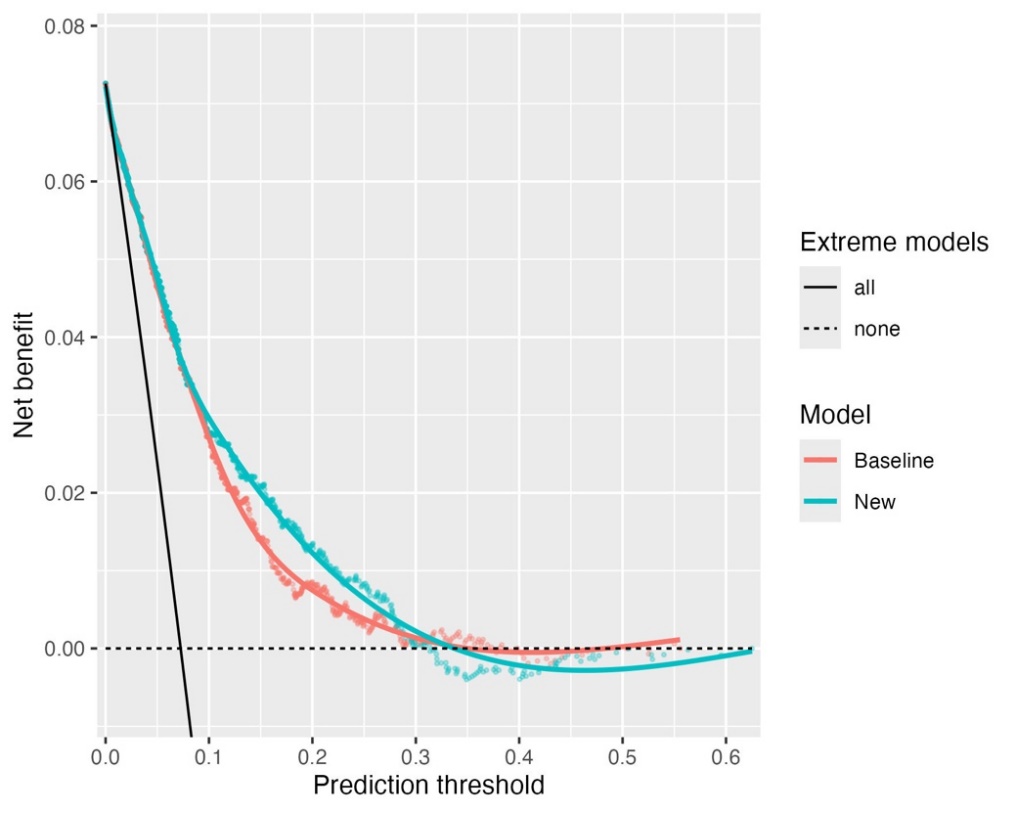


**Outcome:** UAP **Subgroup:** hsTnTMAX < 14

**
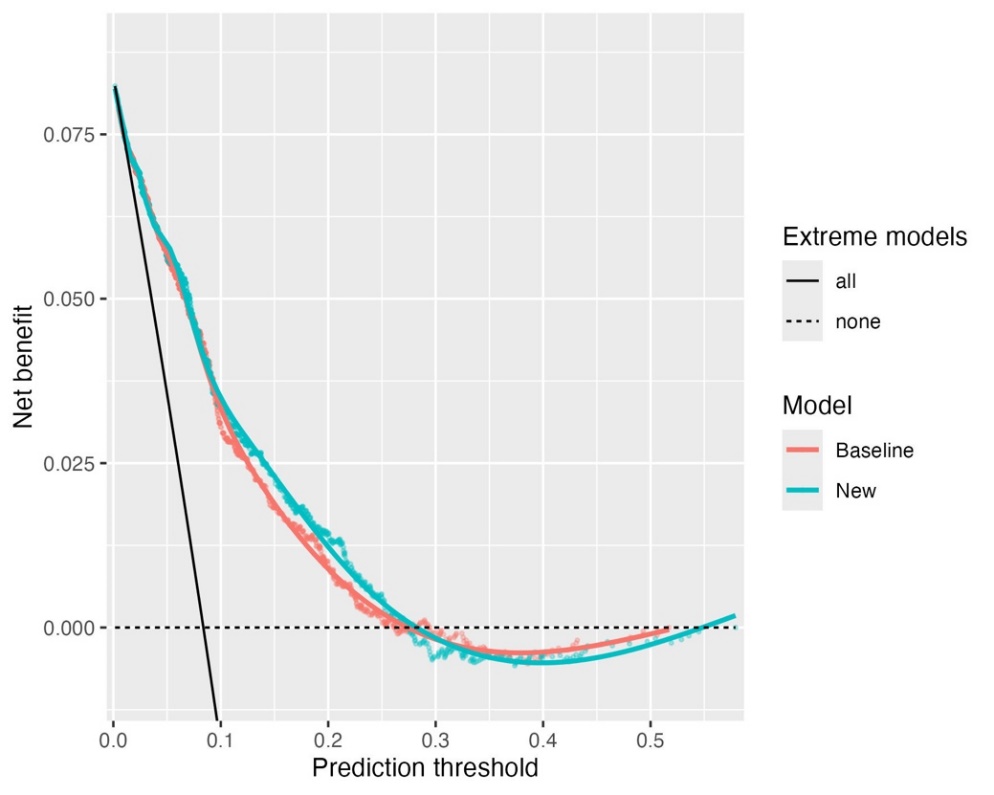
**

**Outcome:** UAP **Subgroup:** hsTnTMAX <14, no HxCAD/MI


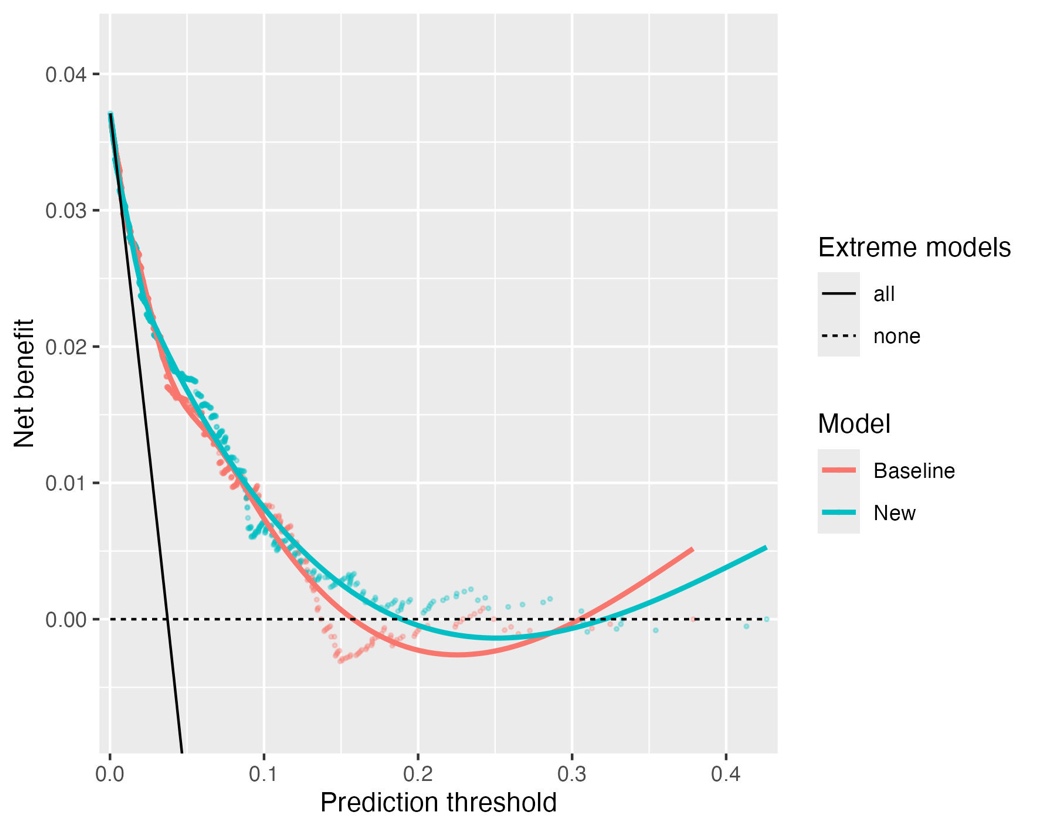


**Outcome:** UAP **Subgroup:** hsTnTMAX >5 and <14


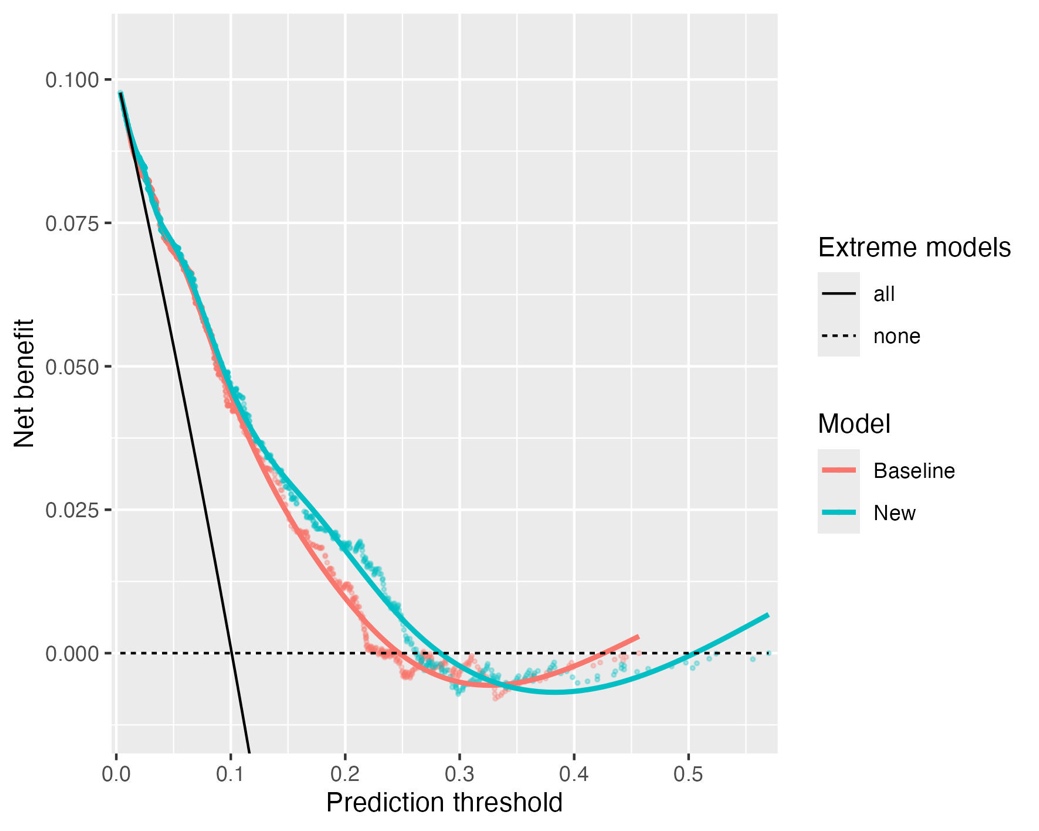


**Outcome:** 70% stenosis **Subgroup:** NOT rule eligible


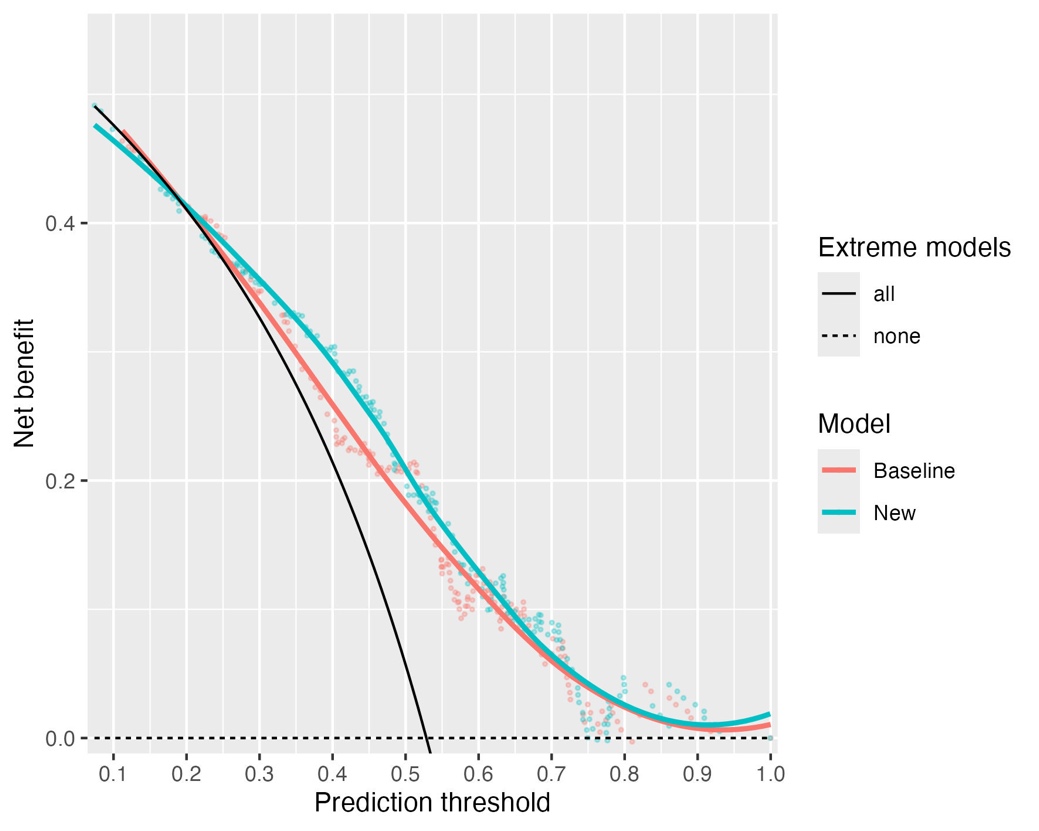


**Outcome:** 70% stenosis **Subgroup:** hsTnTMAX < 14

**
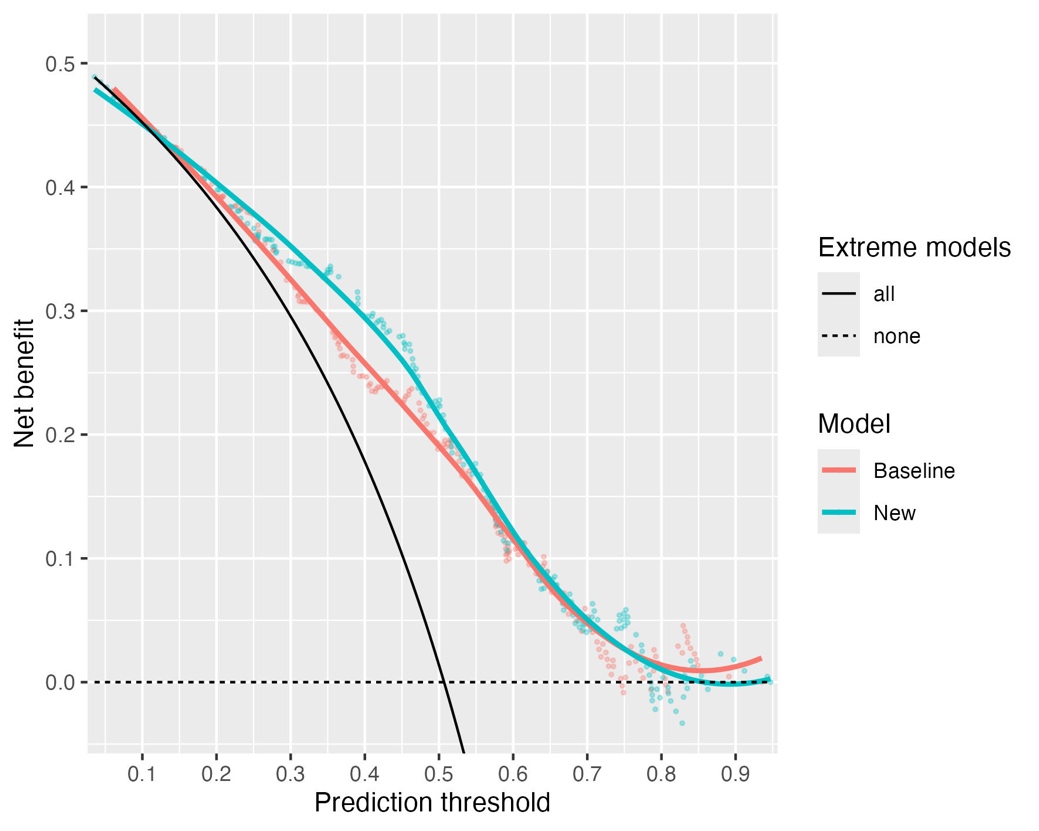
**

**Outcome:** 70% stenosis **Subgroup:** hsTnTMAX <14, no HxCAD/MI


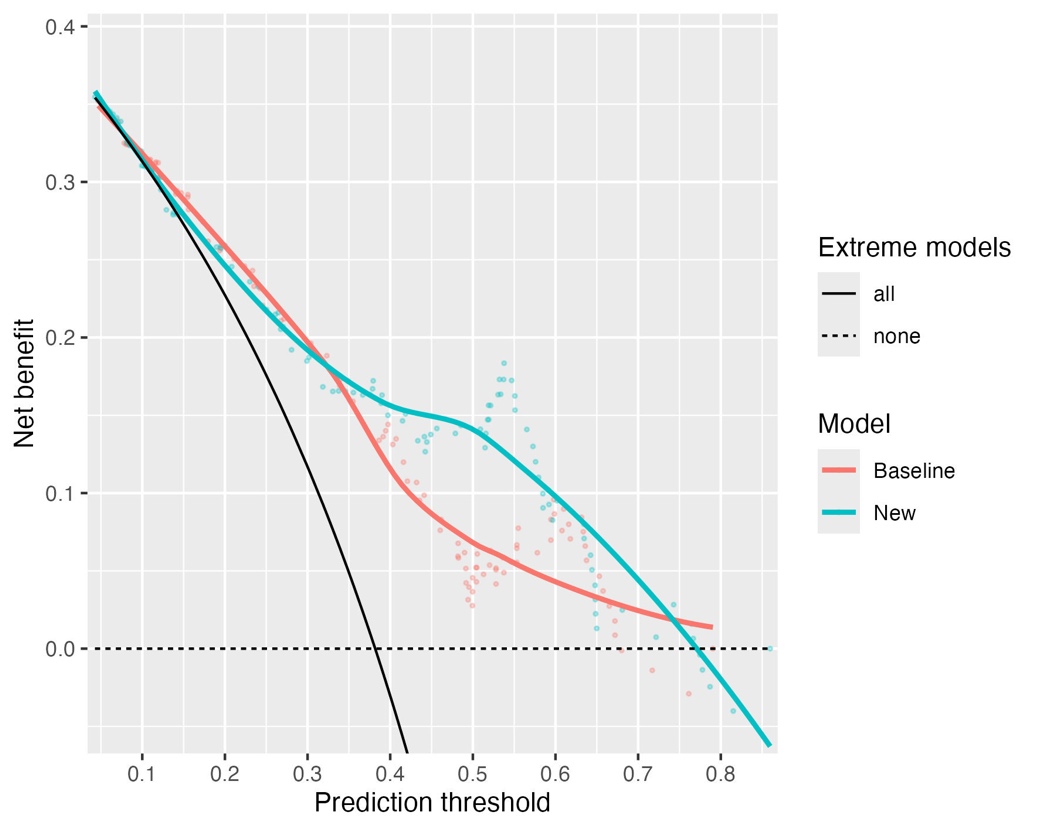


**Outcome:** 70% stenosis **Subgroup:** hsTnTMAX >5 and <14

**
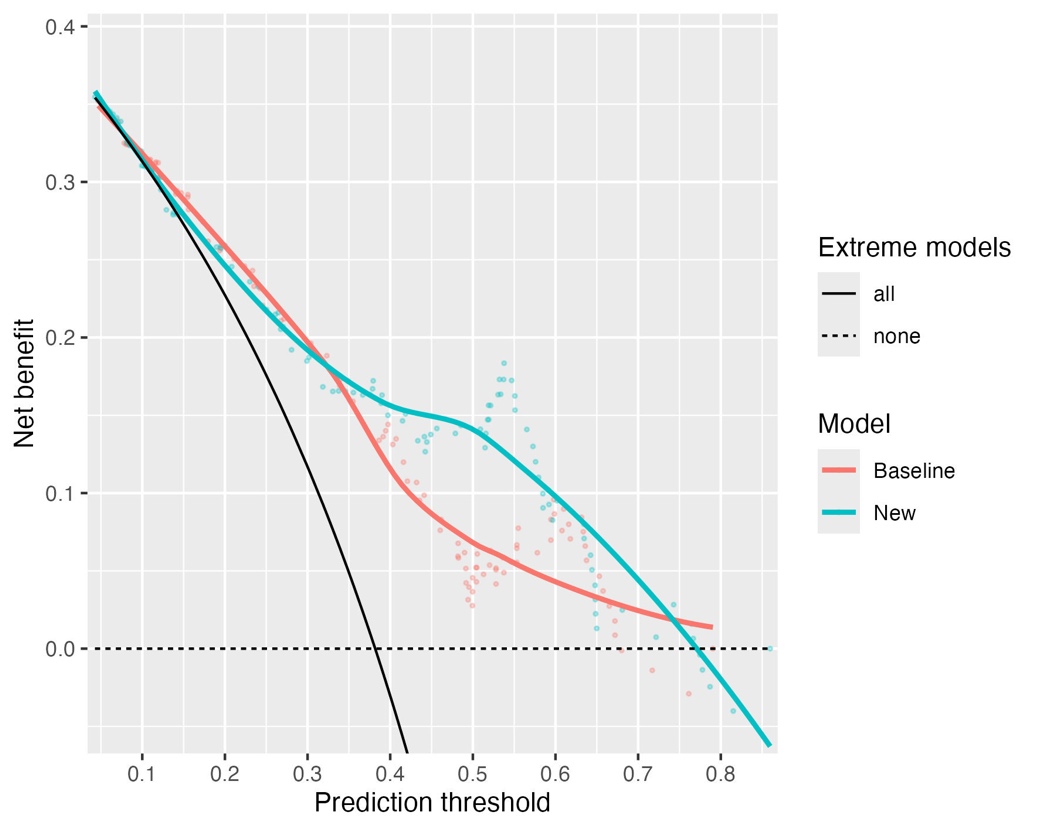
**
